# Hospital Characteristics Associated With Heterogeneity in Institutional Postacute Care Spending Reductions Under the Comprehensive Care for Joint Replacement Model

**DOI:** 10.1001/jamahealthforum.2022.1657

**Published:** 2022-06-17

**Authors:** Thomas H. A. Meath, Cesar Juarez, K. John McConnell, Hyunjee Kim

**Affiliations:** 1 Center for Health Systems Effectiveness, Oregon Health & Science University, Portland

## Abstract

**Importance:**

Prior research concluded that institutional postacute care spending decreased under the Comprehensive Care for Joint Replacement (CJR) model. Less is known about how changes in institutional postacute care spending varied across different types of hospitals.

**Objective:**

To measure hospital-level heterogeneity in the association of the CJR model with changes in institutional postacute care spending and to identify hospital characteristics associated with this variation.

**Design, Setting, and Participants:**

Using 100% Medicare claims data, this cross-sectional study assessed institutional postacute care spending from 2016 to 2017 among US hospitals randomly selected to participate in the CJR model and control group hospitals that were eligible but not selected for the participation in the CJR model. A causal forest was used to estimate the treatment effect of the CJR model conditional on hospital characteristics. Analysis was conducted between October 2019 and October 2021.

**Main Outcomes and Measures:**

The unit of analysis was each hospital; the outcome was the average per-episode Medicare spending for institutional postacute care within 90 days after hospital discharge for hip or knee joint replacement.

**Results:**

This study included 531 CJR participating hospitals and 658 control group hospitals from 2016 to 2017. The CJR model was associated with a $761 reduction in institutional postacute care spending (95% CI, −$1172 to −$351). The reduction in spending under the CJR model did not vary across conditional on hospital characteristics. Limited evidence was found for greater savings among hospitals with higher pre-CJR spending. However, this finding did not hold for hospitals in the highest quintile of pre-CJR spending.

**Conclusions and Relevance:**

In this cross-sectional study of 1189 hospitals, findings did not show strong evidence for significant heterogeneity in how the CJR model was associated with reductions in institutional postacute care spending across a range of hospital characteristics. Savings were not concentrated in hospitals with specific characteristics, such as hospitals with high-volume joint replacement or hospitals serving less medically or socially complex patients. Findings suggest that the CJR model created opportunities for savings across a spectrum of different hospitals.

## Key Points

**Question:**

How were hospital characteristics associated with reductions in institutional postacute care spending under the Comprehensive Care for Joint Replacement (CJR) model?

**Findings:**

This cross-sectional study of 531 CJR participating hospitals and 658 control group hospitals did not find strong evidence for significant heterogeneity in how CJR was associated with reductions in institutional postacute care spending across a range of hospital characteristics.

**Meaning:**

Reductions in institutional postacute care spending under the CJR model did not appear concentrated among a single hospital group or characteristics, suggesting that this payment model created opportunities for savings across a spectrum of different hospitals.

## Teaser

This cross-sectional study measures hospital-level heterogeneity in the association of the Comprehensive Care for Joint Replacement model with changes in postacute care spending and identifies associated characteristics.

## Introduction

In an effort to improve quality and reduce costs, Medicare is moving away from traditional fee-for-service reimbursement to value-based payment models, such as bundled payment models. Value-based payment models provide financial incentives based on the cost and quality of care provided, rather than only the quantity of services provided. Studies of value-based payments have focused on the average effect of the model across the entire population, not the extent to which the effect varied across different types of patients and hospitals. Understanding this heterogeneity is critical for designing future payment models that produce equitable and optimal results across a broad spectrum of patients and hospitals.

The Comprehensive Care for Joint Replacement (CJR) model, Medicare’s first mandatory bundled payment model, provides a unique opportunity to examine the heterogeneous effect of value-based payment models. Starting in April 2016 and continuing until 2024, the CJR model aims to reduce costs and improve the quality of care for patients receiving knee and hip replacement surgeries (hereafter *joint replacements*). The Centers for Medicare & Medicaid Services (CMS) randomly selected 67 metropolitan statistical areas (MSAs) for CJR participation, and most hospitals within those areas were required to participate in the CJR model.^1^ Under CJR, hospitals are accountable for spending incurred during the care episode, including the initial inpatient stay and care provided in the 90 days after hospital discharge. If spending is lower than the benchmark set by CMS, the hospital receives a bonus. Beginning in the CJR model’s second year, hospitals that did not meet the spending benchmark were required to pay a penalty. No specific guidance was provided by CMS on how to reduce spending, allowing for a wide variety of potential approaches.

Prior work concluded that, on average, the CJR model was associated with reduced institutional postacute care spending in its first 2 years.^2,3^ However, only a few studies have examined the potential heterogeneous effects of the CJR model. The official CMS evaluator of the CJR model explored the potential for differences in treatment effects across hospitals with different joint replacement volumes and MSAs with different historical spending and found no evidence of heterogeneity.^3^ Another study found larger reductions in spending among higher-cost hospitals.^4^ However, these evaluations did not attempt to measure more general heterogeneity across hospitals.

Building on this prior work, we used a causal forest approach to assess heterogeneous treatment effects of the CJR model, focusing on institutional postacute care spending, the greatest source of CJR savings.^2,5^ We also examined treatment effect heterogeneity across 4 hospital characteristics: (1) the proportion of medically complex patients undergoing joint replacements, (2) the proportion of socially complex patients undergoing joint replacement patients, (3) each hospital’s number of joint replacements, and (4) the average 90-day institutional postacute care spending for joint replacements prior to the implementation of CJR model.

## Methods

The institutional review board at the Oregon Health & Science University approved this cross-sectional cohort study with a waiver of informed patient consent. This study followed the Strengthening the Reporting of Observational Studies in Epidemiology (STROBE) reporting guideline.^6^

### Sample Selection

Our study included hospitals in 67 MSAs randomly selected by CMS to participate in the CJR model, along with hospitals from 103 control MSAs eligible for the model but not selected to participate.^7^ Our study excluded joint replacement surgeries ineligible for CJR model participation (eg, people with Medicare as a secondary payer and hospitals in the Bundled Payments for Care Improvement initiative for joint replacement). We also excluded surgeries for patients with less than 12 months of enrollment prior to the surgery or less than 3 months following the surgery to ensure sufficient time to capture outcomes and health characteristics. We dropped hospitals that performed fewer than 20 joint replacements during the first 2 years of the CJR model, from April 2016 to October 2017, to obtain stable estimates of institutional postacute care spending. The full sample selection process is presented in the eFigure in the Supplement.

### Data

We used the Medicare Master Beneficiary Summary File and inpatient, skilled nursing facility, home health agency, outpatient, and carrier claims from 2013 through 2017 to identify spending associated with hip or knee joint replacements as well as patient characteristics in the preintervention and postintervention period. We obtained other hospital characteristics from the CMS Provider of Services file during the preintervention period (January 2013 to June 2015; CJR started in April 2016, but CMS officially announced the implementation of CJR in July 2015, and therefore we considered July 2015 to March 2016 as a washout period). We obtained preperiod MSA characteristics from the Area Health Resources File.

### Outcome

Our outcome was the average (per episode) institutional postacute care spending in the 90 days following hospital discharge for joint replacements provided at the hospital, from April 2016 to October 2017. We defined institutional postacute care services as any services provided in a skilled nursing facility, swing bed, inpatient rehabilitation facility, or long-term care hospital.^7^ We adjusted spending for cost of living using the CMS wage index^8^ and for inflation using the Consumer Price Index.^9^

### Hospital Characteristics of Interest

We examined the heterogeneous treatment effect of the CJR model on institutional postacute care spending across the following hospital characteristics, using data from the preintervention period: (1) the proportion of patients undergoing joint replacement who were medically complex, defined as being in the top quartile of Elixhauser readmission risk score,^10^ (2) the proportion of patients undergoing joint replacement who were socially complex, defined as being dually eligible for both Medicaid and Medicare, (3) the joint replacement volume, and (4) the average per-episode institutional postacute care spending in the preintervention period.

We selected these characteristics to capture factors that may be associated with a hospital’s potential to reduce institutional postacute care spending. Hospitals serving a high proportion of medically or socially complex patients may have struggled to reduce institutional postacute care spending because medically or socially complex patients are likely to need more intense postacute care.^11,12^ Hospitals with higher historical institutional postacute care spending may have more room to reduce costs compared with hospitals with lower historical costs. Hospitals providing a low volume of joint replacements will face smaller potential bonuses or penalties under the CJR model and thus may be less incentivized to invest in changes to existing practices.

### Other Hospital and MSA Characteristics

Our models included additional hospital and MSA characteristics measured during the preintervention period to adjust for differences between CJR participating and nonparticipating hospitals that might be associated with institutional postacute care spending. These hospital characteristics included ownership type (nonprofit, for-profit, government, or other), major teaching hospital status (yes/no), the hospital’s operating margin for patient care, number of beds, health system membership status (health system member vs stand-alone hospital), and each hospital’s patient characteristics (joint replacement patients’ mean age, percentage of male patients, percentage of Black patients, percentage of Hispanic patients, percentage of White patients, percentage of patients of other race and ethnicity, percentage of patients with residence in zip codes with a high rate of people with incomes below the federal poverty line, percentage receiving a hip replacement, percentage with a fracture, the mean Elixhauser readmission score, the mean Elixhauser mortality score), and percentage of joint replacement surgeries billed to an external surgeon vs those where the surgeon was employed by the hospital. We identified patient race and ethnicity using the Research Triangle Institute race field in the Master Beneficiary Summary File.^13,14^

We included MSA-level factors: an indicator for the MSA’s sampling strata CMS used during the random sampling for CJR participation; the 2010 MSA population; the Herfindahl–Hirschman Index measuring market competition for Medicare joint replacements^10^; Medicare Advantage penetration rate; proportion of Medicare joint replacements covered by the Bundled Payments for Care Improvement Initiative model; and the number of skilled nursing facility beds and home health agencies per 1000 residents.

### Statistical Analysis

We used causal forests to assess the heterogeneous treatment effect of the CJR model on institutional postacute care spending. Similar to a random forest, a causal forest repeatedly splits the sample into an increasing number of subgroups, producing a series of decision trees leading to subgroups with similar outcomes. Whereas random forests split data to maximize differences in the outcome, causal forests make splitting decisions that maximize differences in the estimated treatment effect. The causal forest allows us to estimate the treatment effect of the CJR model on institutional postacute care spending for each hospital, conditional on its characteristics. In our context, the conditional average treatment effect (CATE) for a particular hospital can be interpreted as the estimated difference in spending between 2 hospitals—one that did participate in the CJR model and one that did not—with a similar set of characteristics.

The causal forest approach has several advantages over regression models, particularly in estimating heterogeneous treatment effects. Unlike regression models that include interaction terms between the treatment indicator and a small set of parameters of interest, the causal forest assesses the heterogeneous treatment effect conditional on a nonlinear function of a rich set of covariates, providing a more complete assessment of potential heterogeneity. Furthermore, causal forests use a 2-part doubly robust estimation, which makes estimates more robust to model misspecification.

The causal forest approach requires making 2 primary assumptions. First, the treatment assignment is unconfounded; that is, the probability of participating in the CJR model conditional on our covariates is independent of the expected outcome conditional on covariates. This assumption is reasonable, as CMS randomly selected MSAs to participate in CJR. Second, our covariates fully capture the features driving heterogeneity in treatment effects. Because the model is unable to detect heterogeneity that is independent of these features, we have included a rich set of covariates in the model.

Following Athey and Wager’s work,^15^ we took the following 2 steps in our causal forest approach. First, we obtained predicted values for our outcome (each hospital’s average institutional postacute care spending) and our treatment (participation in the CJR model). Our outcome predictions were estimated using a standard random forest model based on hospital characteristics, while the treatment predictions came directly from the selection probabilities used by CMS to select MSAs to participate in the CJR model. Second, the causal forests approach uses the predicted outcome and treatment to produce doubly robust estimates of the treatment effect of the CJR model. All models were clustered by MSA because treatment was assigned at the MSA level. Models were weighted by the postperiod joint replacement volume to ensure that our estimated average treatment effect (ATE) is comparable with prior estimates calculated at the patient level.

We plotted the distribution of hospital-level CATE estimates to visually assess the heterogeneity of estimated treatment effects. We also performed 2 formal tests for treatment heterogeneity. First, we split our sample into hospitals above and below the median CATE estimate, calculated an ATE for each subsample, and then tested the difference between the 2 ATEs using a *t* test. Second, we applied the best linear predictor test,^16^ which serves as both a test of model fit and an omnibus test of heterogeneous treatment effects.

To visually examine the heterogeneity across our hospital characteristics of interest, we constructed plots of the ATE and 95% CI stratified by quintiles of each hospital characteristic of our interest. We also formally tested whether each hospital’s characteristic of interest was linearly associated with treatment heterogeneity.^14^ All hypothesis tests were 2-sided, and *P* values < .05 were considered to be statistically significant. Additional modeling details are provided in eAppendix 1 of the Supplement, and the full code used along with raw output are provided in eAppendix 2 of the Supplement. Analyses and data management were conducted between October 2019 and October 2021 using R, version 4.0.3 (R Foundation for Statistical Computing).^17^ Causal forest models were run using the grf package, version 2.0.2.^18^

## Results

Our sample included 531 hospitals participating in CJR (119 002 joint replacement surgeries) and 658 control group hospitals (166 634 surgeries) from April 2016 to October 2017. The Table displays unadjusted hospital and MSA characteristics between CJR participating and control MSAs during the preintervention period. Compared with control group hospitals, CJR participating hospitals provided surgeries to a higher proportion of socially complex patients (10% vs 7%) and a higher proportion of medically complex patients (30% vs 37%). The CJR participating hospitals also had higher historical per-episode spending than control hospitals ($8328 vs $7445). The CJR participating hospitals were located in MSAs with larger populations, greater Medicare Advantage penetration, and greater home health and skilled nursing bed supply. Differences between the CJR and control group hospitals may reflect CMS having oversampled historically high-cost MSAs for participation in CJR.

**Table.** Characteristics of Hospitals in CJR Participating MSAs and Control MSAs During the Preintervention Period From 2012 to 2015

Characteristic	CJR hospitals (n = 531)	Control hospitals (n = 658)	*P* value
**Hospital characteristics**			
Ownership, No. (%)			
For-profit	108 (20.3)	130 (19.8)	.73
Nonprofit	345 (65.0)	440 (66.9)	
Government	66 (12.4)	70 (10.6)	
Other	12 (2.3)	18 (2.7)	
Major teaching hospital, No. (%)	99 (18.6)	119 (18.1)	.86
Operating margins, mean (SD)	2.29 (14.60)	3.23 (17.16)	.32
Bed count, mean (SD)	331.05 (272.55)	300.97 (263.56)	.05
Part of health system, No. (%)	476 (89.6)	590 (89.7)	>.99
Affiliated with PAC facility	78 (14.7)	100 (15.2)	.87
No. of joint replacements, mean (SD)^a^	420.78 (416.11)	466.42 (458.11)	.08
**Joint replacement patient characteristics, mean (SD)**			
Mean age, y	76.33 (1.86)	75.94 (1.77)	<.001
Percent of each sex			
Female	64.84 (5.01)	65.30 (4.82)	.11
Male	34.70 (4.82)	35.16 (5.01)	.11
Mean racial and ethnic %			
Black	5.09 (8.91)	4.71 (6.49)	.42
Hispanic	3.28 (6.25)	4.27 (8.17)	.02
White	87.88 (13.67)	89.08 (13.46)	.13
Other^b^	2.55 (6.24)	3.13 (6.22)	.11
% Socially complex	9.63 (13.02)	6.93 (7.72)	<.001
% Medically complex patient	30.15 (10.25)	27.49 (9.05)	<.001
% Living in high-poverty areas	4.27 (7.55)	3.43 (5.92)	.03
% Hip surgery	47.30 (12.43)	44.72 (12.94)	.001
% Fracture	21.87 (14.74)	19.57 (14.89)	.008
Mean Elixhauser Score			
Readmission	20.49 (5.36)	19.14 (4.78)	<.001
Mortality	5.48 (2.27)	5.01 (2.09)	<.001
Institutional PAC spending, $	8327.89 (4100.30)	7445.32 (3850.51)	<.001
% Billed to external surgeon	97.74 (4.34)	97.74 (4.34)	.98
**MSA characteristics, mean (SD)**			
Population in 2010	4 863 288 (6 337 700)	2 998 120 (2 855 184)	<.001
Joint replacement market competition^c^	1802.18 (1825.93)	2223.65 (1891.68)	<.001
Medicare Advantage penetration percentage	35.32 (12.20)	30.91 (12.23)	<.001
BPCI penetration percentage^d^	14.12 (17.26)	10.08 (12.56)	<.001
No. of home health agencies per 1000 residents	3.35 (2.50)	4.83 (4.06)	<.001
No. of skilled nursing beds per 1000 residents	520.45 (180.73)	499.59 (203.16)	.07

Abbreviations: BPCI, Bundled Payment for Care Improvement; CJR, Comprehensive Care for Joint Replacement model; MSA, metropolitan statistical area; PAC, postacute care.

^a^ Number of CJR-eligible Medicare joint replacement surgery encounters provided by the hospital in the preintervention period.

^b^ Other racial and ethnic group includes patients with Research Triangle Institute race codes indicating Asian/Pacific Islander, American Indian or Alaska Native, other, or unknown.

^c^ Market competition measured as the Herfindahl–Hirschman Index for Medicare joint replacements in the MSA.

^d^ Proportion of Medicare joint replacements covered under the BPCI model.

**Figure 1.**
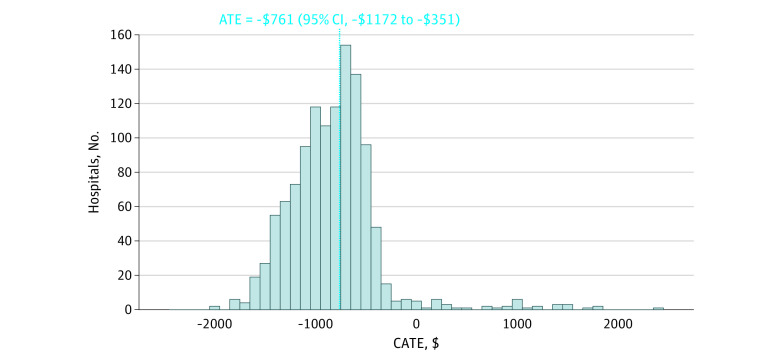
Distribution of Predicted Hospital-Level Conditional Average Treatment Effect (CATE) Estimates (N = 1189) The dashed vertical line shows the overall average treatment effect (ATE).

Figure 1 displays the distribution of individual hospital-level CATE estimates. The dotted line represents the ATE across all hospitals. On average, CJR was associated with decreased institutional postacute care spending of $761 (95% CI, −$1172 to −$351) per joint replacement. Hospital-specific estimates ranged from a reduction of $2025 to an increase of $2421. Although this represents a wide distribution, 97% of the estimates were below zero, suggesting that only a small percentage of hospitals would have experienced an increase in institutional postacute care spending under the CJR model.

We did not find a statistically significant difference in the ATEs when testing for differences between hospitals above vs below the median CATE (difference = $640; 95% CI, −$152 to $1439), although the wide CIs suggested limited precision in our estimates. The lower bound of the CI reflects a scenario in which there was almost no difference in treatment effects across hospitals, while the upper bound would suggest significant heterogeneity—almost twice the size of the overall ATE. The best linear prediction test indicated that the model was well calibrated to estimate the ATE but also found insufficient evidence to suggest treatment heterogeneity (differential prediction coefficient, 1.15; 95% CI, 0.42-1.89; *P* = .06; additional supporting data in eAppendices 1 and 2 in the Supplement).

**Figure 2.**
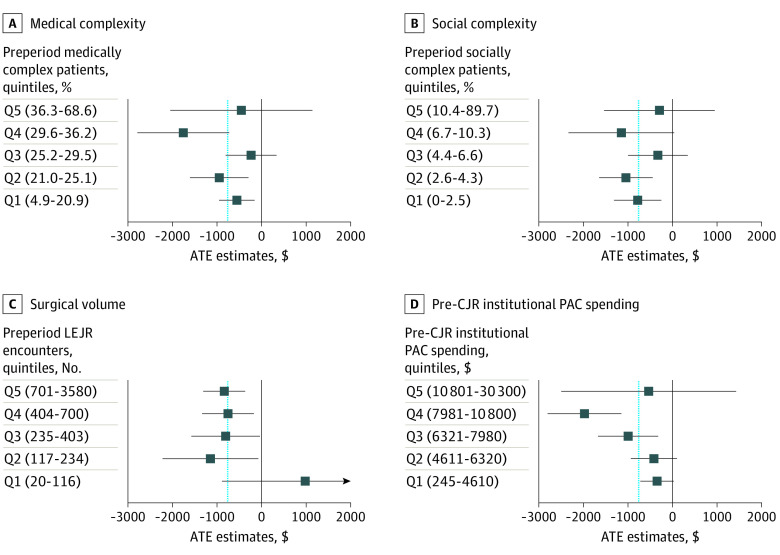
Average Treatment Effect (ATE) and 95% CI Stratified by Quintiles of Hospital Characteristics Each point estimate and error bar represent the ATE and 95% CI among hospitals that fall into a particular quintile of the predictor variable, with quintile 1 capturing the lowest quintile of hospitals and quintile 5 capturing the highest quintile. Quintile ranges are shown in the parentheses for each row. The dashed line shows the ATE across all quintiles. Medical complexity (A) was measured as the proportion of patients undergoing joint replacement who were in the top quartile of the Elixhauser readmission score, social complexity (B) was measured as the proportion of patients undergoing joint replacement who were dually enrolled in both Medicaid and Medicare, surgical volume (C) measured the number of eligible Medicare lower-extremity joint replacement (LEJR) surgeries provided by the hospital, and pre–Comprehensive Care for Joint Replacement model (CJR) institutional postacute care (PAC) spending (D) measured the hospital’s mean per-episode spending for care in an institutional PAC setting.

Figure 2 displays the ATE and 95% CI stratified by quintiles of our 4 hospital characteristics of interest. They showed no clear patterns of association between each hospital characteristic and the treatment effect, with 1 exception. Hospitals in the lowest quintile of pre-CJR institutional postacute care spending had an ATE of −$352 (about 11% reduction from their historical spending), while hospitals in the second highest quintile had an ATE of −$1986 (about 20% reduction from their historical spending). However, we did not observe a similar larger spending reduction among hospitals in the highest quintile of pre-CJR institutional postacute care spending. The CIs for these estimates were wide, suggesting limited power to identify treatment effects among smaller subsets of hospitals. Best linear projection tests did not conclude that any hospital characteristics of interest were linearly associated with treatment heterogeneity.

## Discussion

In this study, we applied causal forest models to investigate the heterogeneous treatment effect of the CJR model on institutional postacute care spending. We found that, on average, the CJR model was associated with a $761 decrease in institutional postacute care spending, similar in size to those found by other studies using a difference-in-differences approach.^2,3^ We found insufficient evidence to suggest that the treatment effect of the CJR model differed across hospitals.

Assessing treatment effects by hospital characteristics highlighted some differences in the ATE across hospitals with different pre-CJR institutional postacute care spending. Hospitals in the fourth quintile of pre-CJR institutional postacute care spending were estimated to generate larger savings than those in the lowest quintile. However, this pattern did not hold for hospitals in the highest quintile of pre-CJR spending.

Our findings suggest that hospitals with higher pre-CJR spending may have had more room to reduce spending than hospitals with relatively low pre-CJR spending. Consistent with this finding, a previous study also found that hospitals whose historical spending was higher than the regional average spending were more likely to reduce institutional postacute care spending under CJR.^4^ Despite potentially greater reductions in institutional postacute care spending, other work has found that higher-cost hospitals were less likely to receive any bonus under the CJR model in 2017^19^ and more likely to leave the CJR model in 2018 when participation became voluntary.^20^

We hypothesized that hospitals with high percentages of medically or socially complex patients may have struggled more to save institutional postacute care spending under CJR. However, we found no evidence for heterogeneity in treatment effects across hospitals with different percentages of medically or socially complex patients undergoing joint replacement. Nevertheless, prior work has consistently shown that hospitals serving a higher proportion of medically or socially complex patients were more likely to be penalized under the CJR model.^19,21^

This may be a result of how CMS set spending benchmarks for the CJR model, which did not adjust for patients’ medical or social complexity. As a result, hospitals serving more complex (and therefore more expensive) patient populations were required to reduce savings by much more than other hospitals, to receive a bonus payment.^21^ When CMS transitioned 33 of the MSAs originally mandated to participate in the CJR model to a voluntary model in 2018, hospitals serving a higher proportion of medically and socially complex patients were more likely to leave the CJR model,^20^ even though these hospitals had patients who may have benefited the most from care coordination efforts and may have generated the largest savings. Fortunately, as part of the extension of the CJR model through 2021 to 2024, CMS updated the benchmark spending algorithm to adjust for patient age, number of chronic conditions (as measured by the CMS Hierarchical Condition Categories used to set reimbursement for Medicare Advantage plans), and dual-enrollment status in Medicaid and Medicare.^22^

### Limitations

This study has limitations. First, we examined data from the first 2 years of the CJR model. Our results may not generalize to later years. Second, we only assessed cross-sectional differences in institutional postacute care spending between CJR participating and control MSAs. Third, we clustered our model at the MSA level, but this clustering may have limited the precision of our hospital-level estimates. Fourth, we were unable to assess heterogeneity driven by factors outside our measured hospital characteristics. Finally, our estimates of treatment heterogeneity had wide CIs, suggesting that our model may lack the power necessary to identify more modest treatment heterogeneity.

## Conclusions

Using a nonparametric, machine-learning approach, this cross-sectional study did not find strong evidence for significant heterogeneity in how CJR was associated with reductions in institutional postacute care spending across a range of hospital characteristics. Savings were not concentrated in one grouping of hospitals (eg, hospitals with high-volume joint replacements or hospitals serving a high proportion of patients with less medical or social complexity). Our findings suggest that the CJR model created opportunities for savings across a spectrum of different hospitals.

## Notes

Supplement. **eFigure.** Sample Selection **eAppendix 1.** Additional Methodology **eAppendix 2.** Analysis Code and Documentation
