# Supplementary material for: Hospital Characteristics Associated With Heterogeneity in Institutional Postacute Care Spending Reductions Under the Comprehensive Care for Joint Replacement Model
Source: JAMA Health Forum. 2022 Jun 17;3(6):e221657. doi: 10.1001/jamahealthforum.2022.1657 (PMC9206192; doi:10.1001/jamahealthforum.2022.1657)
Supplement: Supplement. — eFigure. Sample Selection eAppendix 1. Additional Methodology eAppendix 2. Analysis Code and Documentation [file jamahealthforum-e221657-s001.pdf]

Supplemental Online Content

Meath THA, Juarez C, McConnell KJ, Kim H. Hospital characteristics associated with heterogeneity in institutional postacute care spending reductions under the Comprehensive Care for Joint Replacement model. *JAMA Health Forum.* 2022;3(6):e221657. doi:10.1001/jamahealthforum.2022.1657

eFigure. Sample Selection

eAppendix 1. Additional Methodology

eAppendix 2. Analysis Code and Documentation

This supplemental material has been provided by the authors to give readers additional information about their work.

© 2022 Meath THA et al. *JAMA Health Forum.*

eFigure. Sample Selection

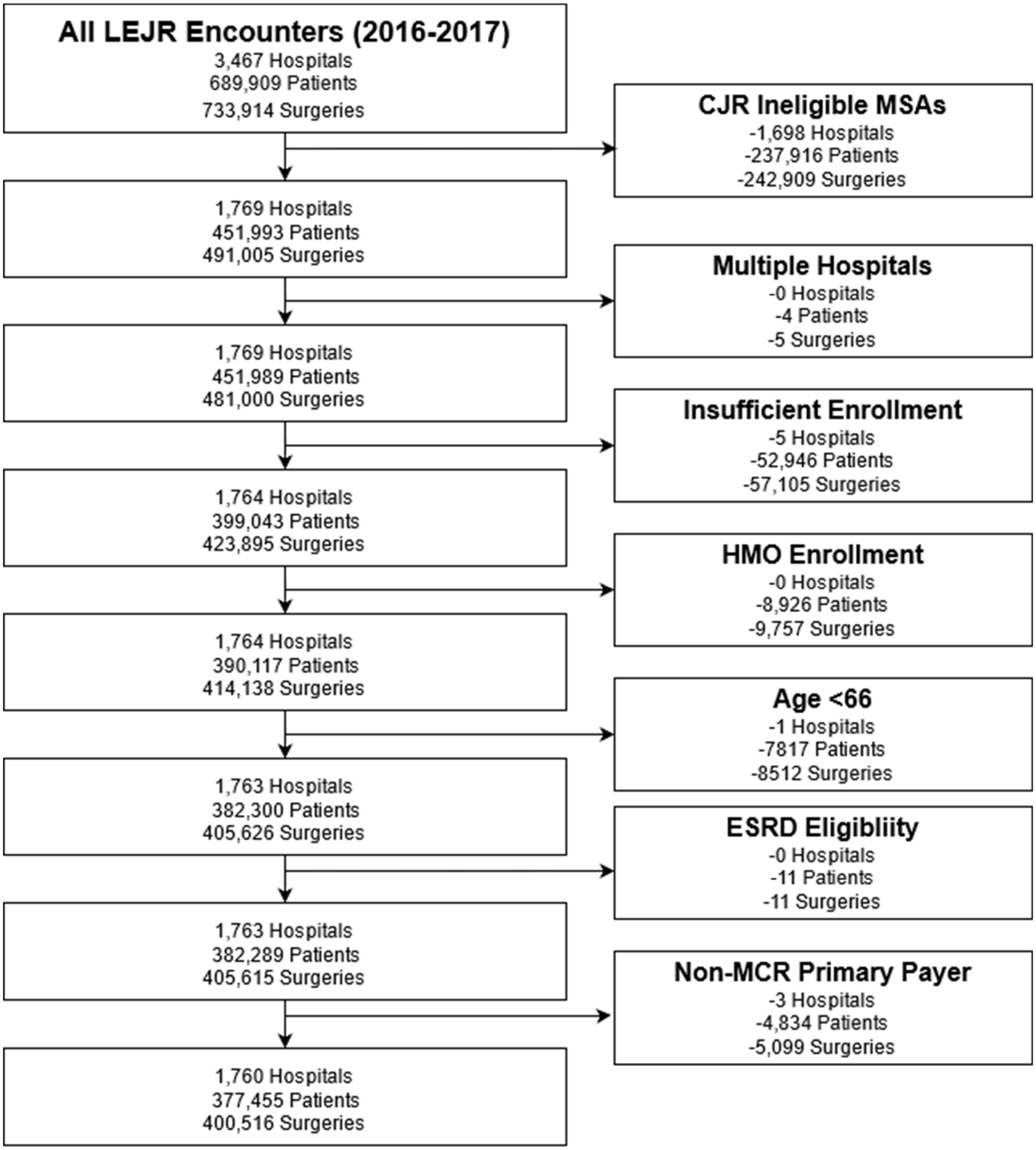

© 2022 Meath THA et al. *JAMA Health Forum.*

Continued:

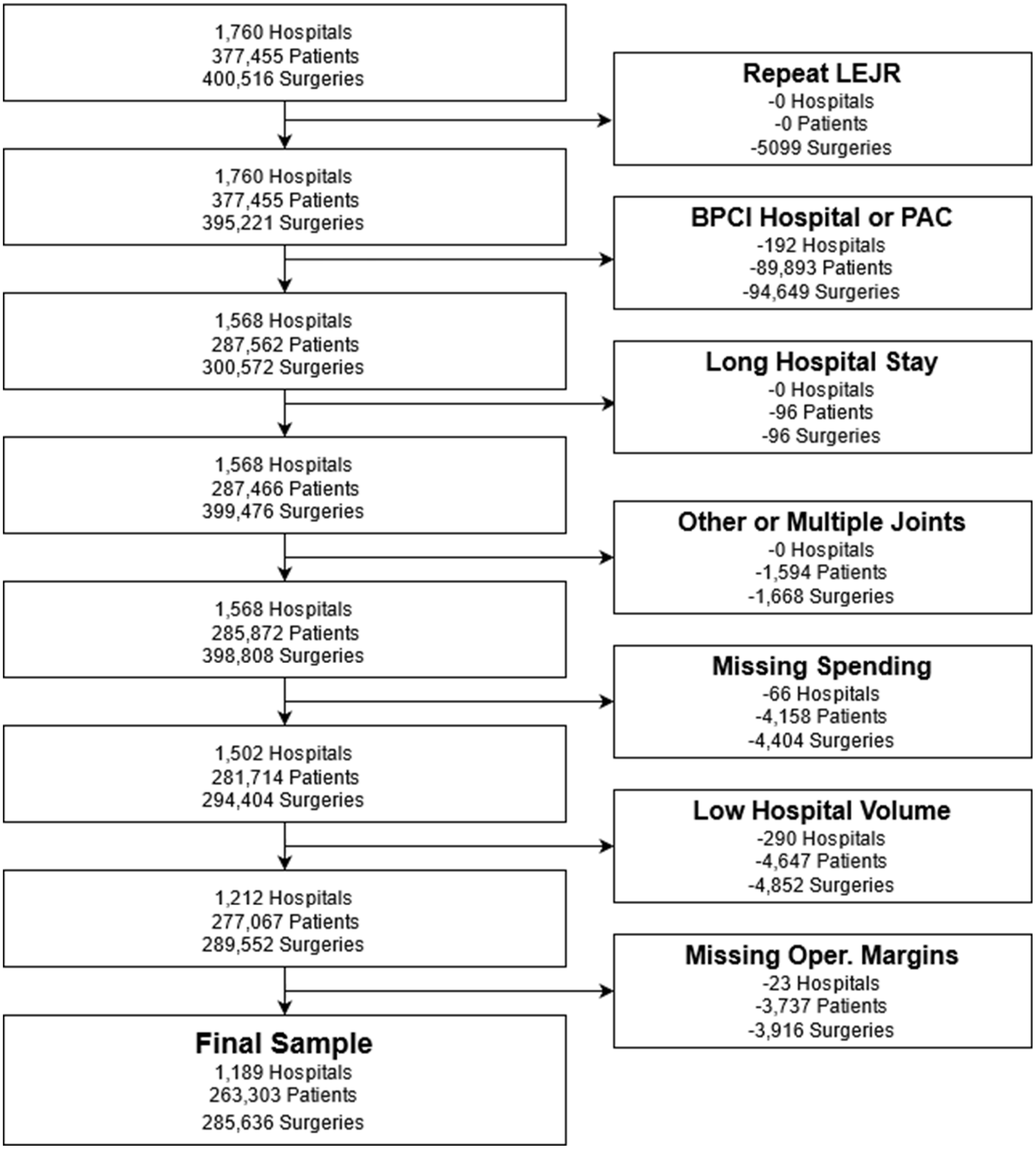

© 2022 Meath THA et al. *JAMA Health Forum.*

eAppendix 1. Additional Methodology

Our studied used causal forests using the `grf` R package version 2.0.2 (Tibshirani et al., 2021) to assess the heterogeneous treatment effect of CJR model on institutional post-acute care spending. Causal forests are type of generalized random forest described in Wager & Athey (2018). Generalized random forests in turn are extension of the common random forest machine learning model (Athey et al., 2019).

Theoretical framework

Random forests are a type of ensemble machine learning that seek to predict an outcome *y* using a set of predictor features *x.* Random forests average over a large number of classification and regression trees, which are very simple non-parametric prediction models that attempt to split the data into recursive partitions in such a way to maximize differences in the outcome between subgroups. The final subgroups in the tree are referred to as the leaves, and hopefully identify distinct subpopulations predictive of the outcome. This type of model is often referred to as "greedy" in the sense that they will leverage random variance unique to the sample to predict in a way that may not be generalizable to other samples. To avoid this, it is common to split the data into two samples for each tree, the training sample that is used to build the tree and the test sample which is used to predict. In our sample we also apply a "honest" correction which further splits the training sample into one subsample that is used to identify the splitting rules for the tree and another that estimates the predictions for each leaf. Each tree in the forest also each only consider a subset of the covariates so that they do not all identify the same set of subgroups. The final prediction for each observation is the average of predictions for all trees where the observation was not part of the training sample, also referred to as "out of bag predictions" because the observation was not part of the "bag" used to create the trees.

Unlike random forest, generalized random forests are optimized to split based on something other than simply maximizing the differences in the outcome. In the case of the causal forests we use, the forests split instead to maximize differences in the treatment effect. This is accomplished using what is called the R Learner objective function described in Nie and Wager (2017), which is an extension of the doubly-robust estimator for treatment effect described in Chernozhukov et al. (2018). The method uses predicted values (or rather the residuals from those predictions) for both the outcome and the treatment to estimate the treatment effect, and is often called doubly robust because the method is robust to misspecifications in either (but not both) of the prediction models. The full conceptual framework for the approach is better described in Wager & Athey (2018).

Conditional Average Treatment Effects (CATE)

For each hospital in our dataset, the causal forest model will estimate a treatment effect conditional on the hospital's characteristics, also called a Conditional Average Treatment Effect (CATE). This can be interpreted as the estimated difference in outcomes between hospitals with similar features that fall into the same leaves based on the splitting rules in the causal forest. The precision of these estimates will depend both on the strength of the underlying heterogeneity, the association between the provided covariates and that heterogeneity, and the amount of observations that share a covariate space.

Running the Causal Forest

In practice, our application of the causal forest method followed a number of distinct steps borrowed from Athey & Wager (2019). First, we predict our outcome (per-episode institutional post-acute care

© 2022 Meath THA et al. *JAMA Health Forum.*

spending) using a random forest fit with our full list of covariates, minus treatment. Second, we estimate the probability of treatment, which in the case of the CJR program is the selection probability used by CMS as part of the stratified random sampling for participation in the CJR program. Next we feed both of those predicted values into a naïve causal forest using the default settings and including all covariates. For each covariate in this model, we measure its importance, or how often it was used as a splitting rule in the forest. We use this measure of importance to drop the covariates in the bottom half of importance. This culling allows the final forest to focus on splitting only the most important covariates. Finally, we run a causal forest using the remaining covariates. This final run also runs a series of smaller forests to tune some of the hyperparameters that guide the fit of the forest, specifically the minimize sample size for leaves and the number of covariates considered in each tree.

All of the models described above are also specified to account for clustering of hospitals within MSAs, both because our treatment was assigned at the MSA level and also because hospitals in the same geographic area often share post-acute care resources. Athey & Wager (2019) found that failing to account for clustering will produce CATE estimations with greater precision that overestimate the heterogeneity in the model. We found similar results and have chosen to use the more conservative clustering approach.

The full code used to run this analysis is presented in the eSection 2 for both transparency and to facilitate replication.

Model Assumptions

The causal forest approach requires two major assumptions and a number of smaller assumptions. First, similar to other estimators of treatment effect it assumes the treatment assignment is exogenous, and thus that the treatment effect is not confounded by unmeasured variables that contribute to both the treatment assignment and the outcome. This assumption is well met in our study because CJR participation was randomly assigned by CMS and is thus independent of any potential confounders. The selection probabilities for each strata of the random sample were provided by the Lewin Group, who were the official evaluators of the CJR model. Second the model assumes that the covariates provided adequately predict heterogeneity. The model is unable to estimate heterogeneity that is independent of the covariates provided. This assumption is not testable, however we have attempted to provide the model with a rich set of patient, hospital and MSA-level covariates.

The model also assumes that the treatment and control groups have sufficient overlap in their probability of receiving treatment (met by the random assignment), and that the probability of receiving treatment is bound within 0,1 (met by the random assignment).

Best Linear Predictor Test

The best linear predictor test serves as both a test of model fit and an omnibus test of whether our model is predicting heterogeneity (Chernozhukov 2017). This test attempts to fit a linear model predicting the treatment effect estimator using two predictors, the mean prediction from the causal forest and the amount each hospital's CATE differs from that mean prediction. A significant coefficient near one for the mean prediction coefficient indicates that the model is accurately capturing the average treatment effect. A significant coefficient near one for the differential coefficient indicates that the model is accurately capturing heterogeneity in the model, while a value of zero implies that the

model did not capture heterogeneity. Thus, the p-value for the differential coefficient can be used as a test of treatment heterogeneity.

The results of the best linear projection test for our model are presented below. This test suggests that our model is accurately capturing the average effect, but that it does not detect significant heterogeneity (though the estimate is relatively close to 1).

	Estimate	Std. Error	t-value	p-value
Mean Forest Prediction	0.968	0.278	3.49	<0.001
Differential Prediction	1.155	0.735	1.57	0.058

Median Test of Treatment Heterogeneity

Another test we used to check for treatment heterogeneity was to split the dataset into two groups, those with CATE estimates above the median CATE and those below the median, and then calculate the Average Treatment Effect for each group. We can then calculate the difference in ATEs and test its significance using a two-sample t-test. This method is suggested in Athey & Wager (2019).

Best Linear Project Test

The best linear projection test examines whether a single predictor variable is linearly associated with treatment effect heterogeneity. It simply predicts the treatment estimator using a linear model including the predictor of interest. This method may not be able to identify non-linear associations, which is why we also examined the ATE and CATE distribution stratified into quintiles of each hospital characteristic of interest.

References

Tibshirani J, Athey S, Sverdrup E, Wager S. *Grf: Generalized Random Forests*.; 2021. https://CRAN.R-project.org/package=grf

Wager S, Athey S. Estimation and Inference of Heterogeneous Treatment Effects using Random Forests. *Journal of the American Statistical Association*. 2018;113(523):1228-1242. doi:10.1080/01621459.2017.1319839

Athey S, Tibshirani J, Wager S. Generalized random forests. *Ann Statist*. 2019;47(2):1148-1178. doi:10.1214/18-AOS1709

Nie X, Wager S. Quasi-Oracle Estimation of Heterogeneous Treatment Effects. *arXiv:171204912 [econ, math, stat]*. Published online August 6, 2020. Accessed April 7, 2022. http://arxiv.org/abs/1712.04912

Chernozhukov V, Chetverikov D, Demirer M, et al. Double/debiased machine learning for treatment and structural parameters. *The Econometrics Journal*. 2018;21(1):C1-C68. doi:10.1111/ectj.12097

Athey S, Wager S. Estimating Treatment Effects with Causal Forests: An Application. *arXiv:190207409 [stat]*. Published online February 20, 2019. Accessed January 23, 2020. http://arxiv.org/abs/1902.07409

Victor Chernozhukov, Mert Demirer, Esther Duflo, Iv'an Fern'andez-Val. *Generic Machine Learning Inference on Heterogenous Treatment Effects in Randomized Experiments*. arXiv.org; 2017. https://ideas.repec.org/p/arx/papers/1712.04802.html

© 2022 Meath THA et al. *JAMA Health Forum.*

eAppendix 2. Analysis Code and Documentation

Differential Treatment of CJR:

Modeling Code

Thomas H.A. Meath

2022-04-08

Table of Contents

Context ... 9

Preliminary Work ... 10

Switches ... 10

Set Seed ... 10

Set paths ... 10

Load Required Packages ... 10

Custom Functions ... 11

Check Overlap ... 13

split_ate ... 14

Fancy Quantile ... 15

Quantile ATE ... 16

Table One ... 17

Structure Data ... 19

Outcomes ... 19

Treatment Variable ... 20

X covariates ... 20

Clusters ... 20

Weights ... 20

Analysis ... 21

Run Model ... 21

Plot CATE ... 22

Test Heterogeneity ... 23

Median Test ... 23

BLP Test ... 23

© 2022 Meath THA et al. *JAMA Health Forum.*

Test Individual Predictors 24
Stored Plot settings 24
Predictor: Complex Patient Percent 24
Predictor: Percent Dual-Eligible 27
Predictor: LEJR Volume 30
Save Output 35
CATE estimates 35
CATE Histogram 35
Ridgelines 35
Stratified ATE 36
Session Info 37

File created by: Thomas Meath, 2020-03-12

© 2022 Meath THA et al. *JAMA Health Forum.*

Context

We are interested in estimating a hospital-level Conditional Average Treatment Effect (CATE), and then looking at the factors associated with different CATE. One way of doing this is using a Causal Forest (CF) or Generalized Random Forest (GRF).

We are going to be following the process described by Susan Athey previously.

Steps:

1. Format data in matrix form
2. Run a simple random forest predicting each outcome using covariates X
3. Run a simple random forest predicting CJR participation using covariates X
4. Run a first pass causal forest using the predictions from *2* and *3*
5. Identify covariates to use in final model
6. Run cross-validated cauasal forest using covariates identified in *5*
7. Estimate the Average Treatment EFfect (ATE) and compare to standard methods
8. Plot histogram of CATEs
9. Test for treatment heterogeneity using median and BLP test
10. Plot stratified density curves for CATE across deciles of variables of interest

© 2022 Meath THA et al. *JAMA Health Forum.*

Preliminary Work

Switches

```
strata_vars <- c("pct_complex_pt", "pct_dual", "lejr_n", "pac_spending
")
```

Set Seed

```
set.seed(8321091)
```

Set paths

```
inpath  <- "/home/groups/chse/WorkingData/ARG_30_NIHJointReplacement/D
ata/analytic_datasets/DiffTx"

codepath <- "/home/groups/chse/WorkingData2/User_Folders/Thomas/active
_projects/CJR_Disparities/Manuscripts/DiffTx/analysis/"

plot_path <-
  file.path("/home/groups/chse/WorkingData/ARG_30_NIHJointReplacement/
Output/Plots/p6_DiffTx",
            format(Sys.Date(), "%Y%m%d"))

tab_path <-
  file.path("/home/groups/chse/WorkingData/ARG_30_NIHJointReplacement/
Output/Tables/p6_DiffTx",
            format(Sys.Date(), "%Y%m%d"))

if(!dir.exists(plot_path)){dir.create(plot_path)}
if(!dir.exists(tab_path)){dir.create(tab_path)}
```

Load Required Packages

```
packs <- c("data.table", #Best package for managing large datasets
           "ggplot2",    #Makes pretty graphics
           "stringr",    #String manipulation
           "ggridges",   #Ridgeline Plots
           "patchwork",  #Combine Plots
           "grf",        #GRF functions
           "dplyr",      #Tidyverse functions
           "scales",     #Scaling functions
           "rpart",      #CART models
           "tableone",   #Table One creation
           "magrittr"    #Enables usf function piping
           )

##
## Attaching package: 'dplyr'
```

© 2022 Meath THA et al. *JAMA Health Forum.*

```
## The following objects are masked from 'package:data.table':
##
##     between, first, last
```

```
## The following objects are masked from 'package:stats':
##
##     filter, lag
```

```
## The following objects are masked from 'package:base':
##
##     intersect, setdiff, setequal, union
```

Custom Functions

. ### run_grf ###

Wrapper for GRF functions.

```
run_grf <- function(outcome, treatment, x_matrix,
                    w_hat = NULL,
                    x_matrix_ps = NULL,
                    cluster     = NULL, weight = NULL,
                    trim_vars   = TRUE,  #If TRUE, this runs the model
using only the top 50% of variables by importance
                    force_vars = NULL,  #Vector of column names to nev
er drop from the model. NULL imposes no restriction.
                    check_ps    = FALSE, #If TRUE, function checks PS m
odel overlap
                    debug       = FALSE, equalize.cluster.weights = FAL
SE){

  #Predict Y~X
  y_hat <-
    regression_forest(X=x_matrix, Y = outcome, clusters = cluster, sam
ple.weights = weight) %>%
    predict %>%
    .$predictions

  #Predict W~X (PS Model), if w_hat not null
  if(is.null(w_hat)){

    if(x_matrix_ps %>% is.null){x_matrix_ps <- x_matrix}

    w_hat <-
      regression_forest(X=x_matrix_ps, Y = treatment,
                        clusters = cluster, sample.weights = weight) %
>%
      predict %>%
```

© 2022 Meath THA et al. *JAMA Health Forum.*

```
      .$predictions
  }

  #Check Overlap if switch is true
  if(check_ps == TRUE){check_overlap(w_hat, treatment) %>% plot}

  #Run Var selection GRF if trim_vars == TRUE
  if(trim_vars == TRUE){

    cf_naive <- causal_forest(Y = outcome, X = x_matrix, W = treatment
,
                              Y.hat = y_hat, W.hat = w_hat,
                              clusters = cluster,
                              sample.weights = weight,
                              equalize.cluster.weights = equalize.clus
ter.weights)
    #Select top 50% important
    naive_var_imp <- variable_importance(cf_naive)
    naive_var_imp <- cbind(naive_var_imp, naive_var_imp > median(naive
_var_imp))
    row.names(naive_var_imp) <- colnames(x_matrix)
    colnames(naive_var_imp) <- c("var_importance", "keep")
    naive_var_imp <- naive_var_imp[order(naive_var_imp[,1]),]

    rownames(naive_var_imp[naive_var_imp[,2] == 0,]) %>%
      sort %>%
      paste0("  -", ., collapse = "\n") %>%
      paste0("Dropped Unimportant Vars:\n", .) %>%
      message

    keep_cols <- c(rownames(naive_var_imp[naive_var_imp[,2] == 1,]), f
orce_vars) %>% unique

    x_matrix <- x_matrix[, keep_cols]
  }

  #Run BCF
  cf_final <- causal_forest(Y = outcome, X = x_matrix, W = treatment,
                            Y.hat = y_hat, W.hat = w_hat, honesty.prun
e.leaves = FALSE,
                            num.trees = 10000,
                            clusters = cluster,
                            sample.weights = weight,
                            equalize.cluster.weights = equalize.cluste
r.weights,
                            tune.parameters = c("mtree", "min.node.siz
```

© 2022 Meath THA et al. *JAMA Health Forum.*

```
e"),
                                                        tune.num.trees = 500,
                                                        tune.num.reps = 100,
                                                        tune.num.draws = 2000)

  return(cf_final)
}
```

Check Overlap

Function to compare the overlap in propensity scores, one of the assumptions

```
check_overlap <-
  function(ps_scores, treatment){
    #make data table
    temp <- data.table(group = treatment[, 1],
                       psvalue = ps_scores)

    plot <-
      temp %>%
      #Factor group
      mutate(group = factor(group,
                            levels = c(1, 0),
                            labels = c("Treatment",
                                       "Control"))) %>%

      #Create bins
      mutate(ps_binned = cut(psvalue,
                             breaks = seq(-0.025, 1, 0.025),
                             include.lowest = T,
                             labels = seq(0, 1, 0.025))) %>%

      #Calculate distribution of bins for each group
      group_by(group) %>%
      mutate(n = n()) %>%
      group_by(group,
               ps_binned) %>%
      summarize(p = n()/max(n)) %>%
      mutate(
        p = case_when(group == "Treatment" ~ p,
                      group == "Control"   ~ -p)
      ) %>%
      #Plot
      ggplot(aes(x = ps_binned,
                 y = p,
                 fill = group)) +
```

© 2022 Meath THA et al. *JAMA Health Forum.*

```
    geom_bar(stat = 'identity',
             width = 0.75,
             position = position_stack()) +

    geom_hline(yintercept = 0, color = "black") +

    geom_text(aes(y = max(p)*1.1,
                  x = "0.5",
                  label = "Treatment"),
              color = "dodgerblue",
              size = 12) +

    geom_text(aes(y = min(p)*1.1,
                  x = "0.5",
                  label = "Control"),
              color = "gray60",
              size = 12) +


    scale_fill_manual(values = c("dodgerblue",
                                 "gray60")) +

    scale_x_discrete(name = "Propensity Score",
                     breaks = seq(0.0, 1, 0.1),
                     limits = seq(0.0, 1, 0.025) %>% as.character,
                     label  = seq(0, 1, 0.1) %>% percent) +

    scale_y_continuous("Density",
                       breaks = 0) +

    theme_minimal() +
    theme(legend.position = "none")

  return(plot)

}
```

split_ate

Splits the dataset into two parts, calculates the ATE for each half, then runs a simple t-test to see if the ATE differ

```
split_ate <- function(model, split){

  if(!is.logical(split)){stop("Split rule must be logical")}

  ATE_true <- average_treatment_effect(model,  subset = split)
```

© 2022 Meath THA et al. *JAMA Health Forum.*

```
  ATE_false <- average_treatment_effect(model,  subset = !split)

  ATE_diff <- c(ATE_true[1] - ATE_false[1],
                sqrt(ATE_true[2]^2 + ATE_false[2]^2))

  result <-
    rbind(data.table(split = "true",  est = ATE_true[1], margin = qnor
m(0.975)*ATE_true[2]),
          data.table(split = "false", est = ATE_false[1], margin = qno
rm(0.975)*ATE_false[2]),
          data.table(split = "diff",  est = ATE_diff[1], margin = qnor
m(0.975)*ATE_diff[2]))

  return(result)

}
```

Fancy Quantile

Creates a fancy quantile label with length equal to the input string. Saves some coding later.

```
fancy_quantile <- function(x, quantiles, label_func = identity){

  #calcualte quantiles and cut
  q_cuts <- quantile(x, quantiles)
  q_pred = cut(x, breaks = q_cuts, include.lowest = T)

  #Calculate Quantile number
  q_lab_pre = cut(x, breaks = q_cuts, include.lowest = T,
                  labels = paste0("Q", 1:(length(q_cuts)-1)))

  #Choose whether to close range with inclusion or exclusion
  # (Brackets or Parens)
  open_type  <- substr(as.character(q_pred), 1,1)
  close_type <- substr(as.character(q_pred),
                       nchar(as.character(q_pred)),
                       nchar(as.character(q_pred)))

  #Calculate Min/Max for each quantile
  q_min <- gsub("[\\(\\[]([0-9\\.e\\+]+),([0-9\\.e\\+]+)\\]",
                "\\1", q_pred) %>%
    as.numeric %>% round(3)

  q_max <- gsub("[\\(\\[]([0-9\\.e\\+]+),([0-9\\.e\\+]+)\\]",
                "\\2", q_pred) %>%
    as.numeric %>% round(3)
```

© 2022 Meath THA et al. *JAMA Health Forum.*

```
  #Build fancy label
  fancy_q <- sprintf("%s %s%s, %s%s",
                     q_lab_pre, open_type,
                     label_func(q_min),
                     label_func(q_max),
                     close_type)

  #Return
  return(fancy_q)

}
```

Quantile ATE

Produces the ATE and 95% CI stratified for quantiles of a single predictor

```
quantiles_ate <- function(model, predictor, quantiles, label_func = id
entity){

  #Get Quantiles
  q_pred <- fancy_quantile(x = predictor, quantiles = quantiles, label
_func = label_func)

  #Frame with row for each quantile group
  output_frame <-
    data.frame(q_label = unique(q_pred), ate_est = 0, ate_low = 0, ate
_high = 0)

  #Iterate over frame to get ATE estimate
  for(i in 1:nrow(output_frame)){
    select_cond <- q_pred == output_frame$q_label[i]
    ate_i <- average_treatment_effect(model, subset = select_cond)

    output_frame$ate_est[i] <- ate_i["estimate"]
    output_frame$ate_low[i] <- ate_i["estimate"] - 1.96*ate_i["std.err
"]
    output_frame$ate_high[i] <- ate_i["estimate"] + 1.96*ate_i["std.er
r"]
  }

  return(output_frame)

}
```

© 2022 Meath THA et al. *JAMA Health Forum.*

#Data Processing#

##Load Data##

```
analytic <-
  file.path(inpath, "analytic_2022-04-06.rds") %>%
  readRDS()
```

Table One

```
analytic2 <-
  analytic[, .(treat, owner_type, maj_teach, op_margin_pat, bed_cnt,
                  in_system, sys_multistate, sys_acutehosp, pac_affil,
                  pac_spending,
                  lejr_n, pct_hip = 100*pct_hip, pct_frac = 100*pct_frac,
                  pct_male = 100*pct_male,
                  pct_black = 100*pct_black, pct_hisp = 100*pct_hisp,
                  pct_oth = 100*pct_oth, pct_white = 100*pct_white, pct_d
ual = 100*pct_dual, pct_poverty = 100*pct_poverty,
                  mean_age, pct_complex_pt = 100*pct_complex_pt, pct_ext_
surg = 100*pct_ext_surg, elix_score_m, elix_score_r,
                  msa_pop_2010, hhi, msa_mcr_adv_pen, msa_hha_rate, msa_s
nf_rate, msa_bpci_pre = 100*msa_bpci_pre)]

vars <- c("owner_type", "maj_teach", "op_margin_pat", "bed_cnt",
          "in_system", "sys_multistate", "sys_acutehosp", "pac_affil",
          "pac_spending",
          "lejr_n", "pct_hip", "pct_frac",
          "pct_male", "pct_black", "pct_hisp", "pct_oth", "pct_white",
"pct_dual", "pct_poverty",
          "mean_age", "pct_complex_pt", "pct_ext_surg", "elix_score_m"
, "elix_score_r",
          "msa_pop_2010", "hhi", "msa_mcr_adv_pen", "msa_hha_rate", "m
sa_snf_rate", "msa_bpci_pre")

vars_cat <- c("owner_type", "maj_teach",
              "in_system", "sys_multistate", "pac_affil")

tab1 <-
  CreateTableOne(vars = vars,
                 strata = "treat",
                 data = analytic2,
                 factorVars = vars_cat)

print(tab1, showAllLevels = TRUE)

##                                Stratified by treat
##                                 level      0
```

© 2022 Meath THA et al. *JAMA Health Forum.*

```
##   n                                                  658
##   owner_type (%)              for-profit        130 (19.8)
##                               non-profit        440 (66.9)
##                               public             70 (10.6)
##                               unknown            18 ( 2.7)
##   maj_teach (%)               0                 539 (81.9)
##                               1                 119 (18.1)
##   op_margin_pat (mean (SD))                    3.23 (17.16)
##   bed_cnt (mean (SD))                        300.97 (263.56)
##   in_system (%)               FALSE              68 (10.3)
##                               TRUE              590 (89.7)
##   sys_multistate (%)          FALSE             388 (59.0)
##                               TRUE              270 (41.0)
##   sys_acutehosp (mean (SD))                   32.78 (50.45)
##   pac_affil (%)               FALSE             558 (84.8)
##                               TRUE              100 (15.2)
##   pac_spending (mean (SD))                  7445.32 (3850.51)
##   lejr_n (mean (SD))                         466.42 (458.11)
##   pct_hip (mean (SD))                         44.72 (12.94)
##   pct_frac (mean (SD))                        19.57 (14.89)
##   pct_male (mean (SD))                        35.16 (5.01)
##   pct_black (mean (SD))                        5.09 (8.91)
##   pct_hisp (mean (SD))                         3.28 (6.25)
##   pct_oth (mean (SD))                          2.55 (6.24)
##   pct_white (mean (SD))                       89.08 (13.46)
##   pct_dual (mean (SD))                         6.93 (7.72)
##   pct_poverty (mean (SD))                      3.43 (5.92)
##   mean_age (mean (SD))                        75.94 (1.77)
##   pct_complex_pt (mean (SD))                  27.49 (9.05)
##   pct_ext_surg (mean (SD))                    97.74 (4.34)
##   elix_score_m (mean (SD))                     5.01 (2.09)
##   elix_score_r (mean (SD))                    19.14 (4.78)
##   msa_pop_2010 (mean (SD))               2998120.14 (2855183.90)
##   hhi (mean (SD))                           2223.65 (1891.68)
##   msa_mcr_adv_pen (mean (SD))                 30.91 (12.23)
##   msa_hha_rate (mean (SD))                     4.83 (4.06)
##   msa_snf_rate (mean (SD))                   499.59 (203.16)
##   msa_bpci_pre (mean (SD))                    10.08 (12.56)
##                              Stratified by treat
##                              1                        p      test
##   n                                531
##   owner_type (%)                   108 (20.3)          0.730
##                                    345 (65.0)
##                                     66 (12.4)
##                                     12 ( 2.3)
##   maj_teach (%)                    432 (81.4)          0.863
```

© 2022 Meath THA et al. *JAMA Health Forum.*

```
##                                          99 (18.6)
##    op_margin_pat (mean (SD))          2.29 (14.60)       0.316
##    bed_cnt (mean (SD))              331.05 (272.55)      0.054
##    in_system (%)                        55 (10.4)        1.000
##                                        476 (89.6)
##    sys_multistate (%)                  264 (49.7)        0.002
##                                        267 (50.3)
##    sys_acutehosp (mean (SD))         41.14 (58.00)       0.008
##    pac_affil (%)                       453 (85.3)        0.871
##                                         78 (14.7)
##    pac_spending (mean (SD))        8327.89 (4100.30)    <0.001
##    lejr_n (mean (SD))               420.78 (416.11)      0.076
##    pct_hip (mean (SD))               47.30 (12.43)       0.001
##    pct_frac (mean (SD))              21.87 (14.74)       0.008
##    pct_male (mean (SD))              34.70 (4.82)        0.109
##    pct_black (mean (SD))              4.71 (6.49)        0.418
##    pct_hisp (mean (SD))               4.27 (8.17)        0.018
##    pct_oth (mean (SD))                3.13 (6.22)        0.108
##    pct_white (mean (SD))             87.88 (13.67)       0.129
##    pct_dual (mean (SD))               9.63 (13.02)      <0.001
##    pct_poverty (mean (SD))            4.27 (7.55)        0.032
##    mean_age (mean (SD))              76.33 (1.86)       <0.001
##    pct_complex_pt (mean (SD))        30.15 (10.25)      <0.001
##    pct_ext_surg (mean (SD))          97.74 (4.34)        0.983
##    elix_score_m (mean (SD))           5.48 (2.27)       <0.001
##    elix_score_r (mean (SD))          20.49 (5.36)       <0.001
##    msa_pop_2010 (mean (SD))     4863288.00 (6337700.35) <0.001
##    hhi (mean (SD))                 1802.18 (1825.93)    <0.001
##    msa_mcr_adv_pen (mean (SD))       35.32 (12.20)      <0.001
##    msa_hha_rate (mean (SD))           3.35 (2.50)       <0.001
##    msa_snf_rate (mean (SD))         520.45 (180.73)      0.065
##    msa_bpci_pre (mean (SD))          14.12 (17.26)      <0.001
```

Save it too

```
print(tab1, showAllLevels = TRUE, printToggle = FALSE) %>%
  write.csv(file = file.path(tab_path, "Table1.csv"),
            row.names = TRUE)
```

Structure Data

We want our outcomes, predictors and adjustment variables all to be separate matrices. I also want to split factors into their dummy variables.

Outcomes

Institutional Discharge

© 2022 Meath THA et al. *JAMA Health Forum.*

```
pac_spending <-
  analytic$pac_spending_post
```

Treatment Variable

```
treatment <-
  analytic[, .(treat)] %>%
  as.matrix
```

X covariates

One version with MSA factors

```
analytic[, msa_group := factor(msa_group)]

ps_vars <- c("msa_group", "in_system",  "pac_spending",
             "hhi", "msa_mcr_adv_pen", "msa_hha_rate", "msa_snf_rate",
             "msa_pop_2010", "msa_bpci_pre",
             "owner_type", "pac_affil", "bed_cnt", "maj_teach", "op_ma
rgin_pat",
             "lejr_n", "pct_hip", "pct_frac", "pct_comp",
             "pct_male", "pct_black", "pct_hisp", "pct_oth", "pct_whit
e", "pct_dual", "pct_poverty", "mean_age",
             "pct_complex_pt", "pct_ext_surg",
             "elix_score_m", "elix_score_r")

x_frame <-
  analytic[, ps_vars, with = FALSE]

x <- model.matrix(~., data = x_frame)
x <- x[, -1]
```

Clusters

We cluster our sampling on MSA (since MSAs were CMS's sampling unit, and there is no treatment variation within MSA)

```
msa <- analytic$msa %>% as.numeric()
```

Weights

Weights are the combination of sample weights and LEJR counts (to match the patient level ATE)

```
weight <- analytic$lejr_n_post
```

© 2022 Meath THA et al. *JAMA Health Forum.*

Analysis

Run Model

```
pac_spending_cf <- run_grf(outcome = pac_spending, treatment = treatme
nt,
                           x_matrix = x, w_hat = analytic$treat_p,
                           force_vars = strata_vars,
                           weight = weight,
                           cluster = msa, check_ps = TRUE)

## `summarise()` has grouped output by 'group'. You can override using
the
## `.groups` argument.
## Dropped Unimportant Vars: -bed_cnt -in_systemTRUE -lejr_n -maj_teac
h
## -msa_bpci_pre -msa_group2 -msa_group3 -msa_group4 -msa_group5 -msa_
group6
## -msa_group7 -msa_group8 -owner_typenon-profit -owner_typepublic
## -owner_typeunknown -pac_affilTRUE -pct_dual -pct_hip -pct_white
```

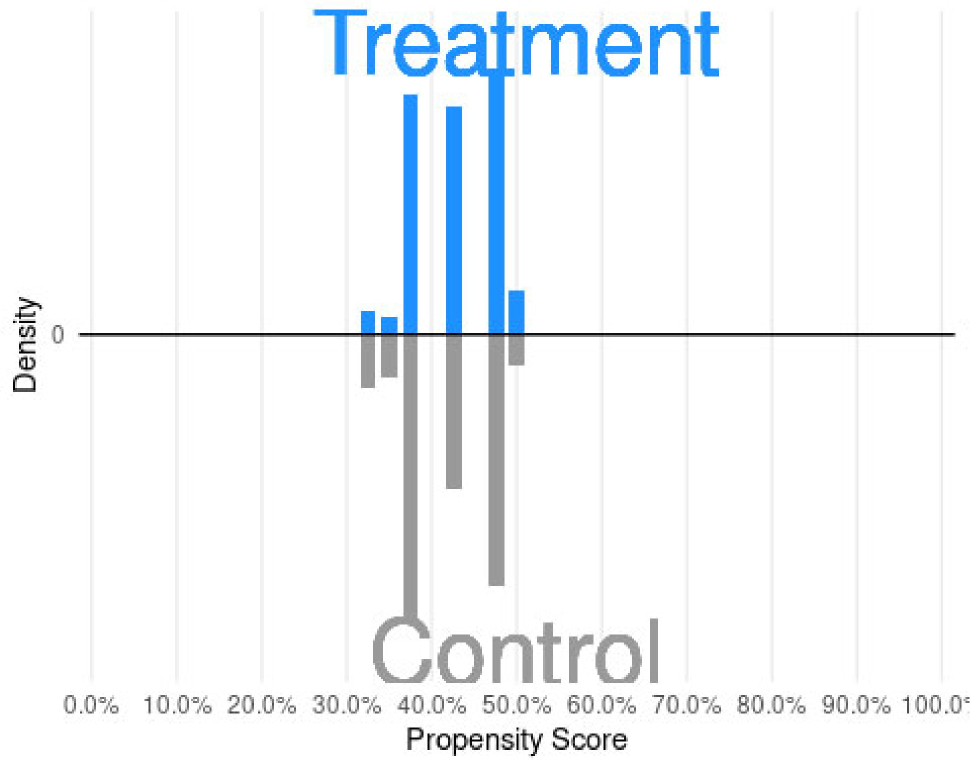

Save CATE

```
analytic[, pac_cate := predict(pac_spending_cf)$predictions]
med_cate <- median(analytic$pac_cate)
```

© 2022 Meath THA et al. *JAMA Health Forum.*

Plot CATE

```
ate <- average_treatment_effect(pac_spending_cf)
ate_est <- ate[["estimate"]]
ate_se  <- ate[["std.err"]]
ate_low <- ate_est - 1.96*ate_se
ate_high <- ate_est + 1.96*ate_se

ate_label <-
  sprintf("ATE = %s (%s, %s)",
          dollar_format(1)(ate_est),
          round(ate_low),
          round(ate_high))

plot_cate <-
  analytic %>%
  ggplot(aes(x = pac_cate)) +
  geom_histogram(binwidth = 100) +
  geom_vline(xintercept = ate_est,
             color = "red", linetype = 2,
             size = 1) +
  annotate("text",
           x = ate_est, y = 60, label = ate_label,
           size = 4,
           color = "white", angle = 90, vjust = -1) +
  scale_x_continuous("CATE ($)", labels = dollar, limits = c(-2500,250
0)) +
  scale_y_continuous("Hospitals (N)", labels = comma) +
  theme_minimal() +
  theme(axis.title = element_text(size = 14),
        axis.text = element_text(size = 12))

plot_cate

## Warning: Removed 2 rows containing missing values (geom_bar).
```

© 2022 Meath THA et al. *JAMA Health Forum.*

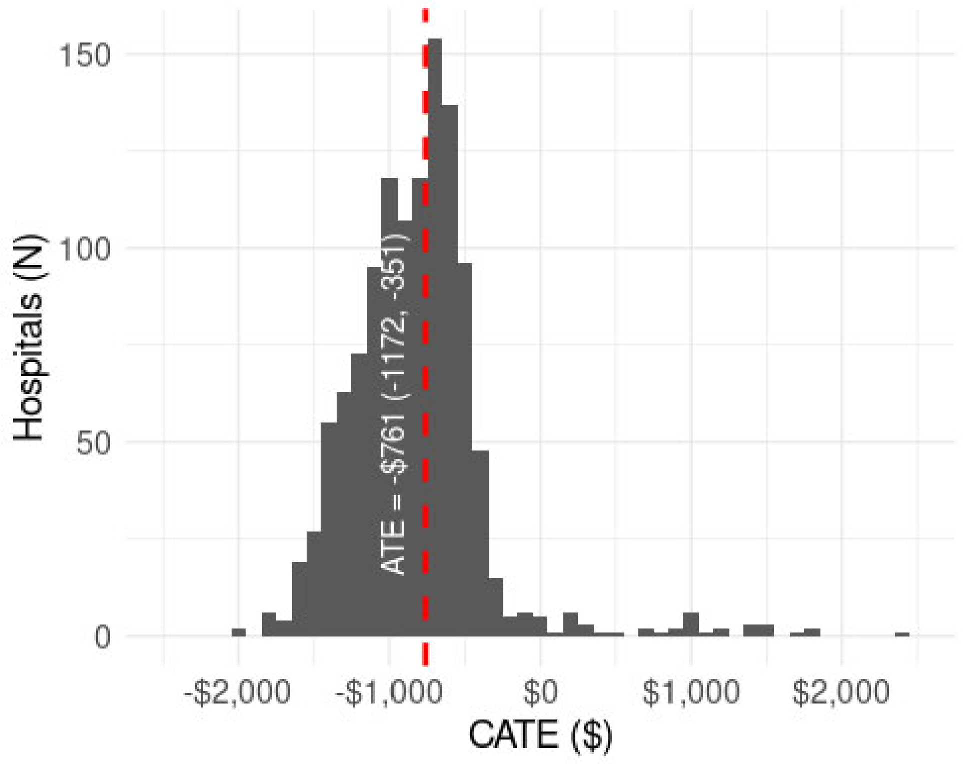

Test Heterogeneity

Median Test6

```
split_ate(pac_spending_cf, analytic$pac_cate > med_cate)
```

split	est	margin
true	-524.2139	425.5810
false	-1164.0412	667.9623
diff	639.8273	792.0182

BLP Test

```
test_calibration(pac_spending_cf)

##
## Best linear fit using forest predictions (on held-out data)
## as well as the mean forest prediction as regressors, along
## with one-sided heteroskedasticity-robust (HC3) SEs:
##
##                                Estimate Std. Error t value    Pr(>t
)
## mean.forest.prediction          0.96801    0.27752  3.4881 0.000252
1 ***
## differential.forest.prediction  1.15459    0.73501  1.5709 0.058242
0 .
```

© 2022 Meath THA et al. *JAMA Health Forum.*

```
## ---
## Signif. codes:  0 '***' 0.001 '**' 0.01 '*' 0.05 '.' 0.1 ' ' 1
```

Test Individual Predictors

Stored Plot settings

Savings some plot settings here for easier swapping later,

First, theme:

```
my_theme <-
  theme_minimal() +
  theme(panel.grid.major.y = element_blank(),
        legend.position = "none",
        plot.title = element_text(size = 16),
        plot.title.position = "plot",
        axis.title.y = element_text(margin = margin(r = 10),
                                    size = 14),
        axis.title.x = element_text(margin = margin(t = 10),
                                    size = 14),
        axis.text.x = element_text(size = 12),
        axis.text.y = element_text(size = 12),
        axis.ticks = element_line(),
        axis.ticks.length.y = unit(5, "mm")
        )
```

Stored values

```
cate_breaks <- seq(-2000, 2000, 1000) #CATE breakpoints
ate_breaks <- seq(-3000, 2000, 1000)
```

Predictor: Complex Patient Percent

Proportion of patients in the top 25% of the Elixhauser readmission risk score.

Best Linear Projection

Test if predictor is linearly associated with DR scores (i.e. Treatment effect)

```
best_linear_projection(pac_spending_cf, analytic$pct_complex_pt)

##
## Best linear projection of the conditional average treatment effect.
## Confidence intervals are cluster- and heteroskedasticity-robust (HC
3):
##
##               Estimate Std. Error t value Pr(>|t|)
```

© 2022 Meath THA et al. *JAMA Health Forum.*

```
## (Intercept)  -837.88     726.83 -1.1528   0.2492
## A1            302.07    3149.65  0.0959   0.9236
```

CATE Ridgeline by Quintile

```
analytic[, complex_pt_q := fancy_quantile(pct_complex_pt,
                                          quantiles = seq(0, 1, 0.2),
                                          label_func = percent_format(
0.1))]
```

Next, the ridgeline plot

```
complex_pat_ridge <-
  analytic %>%
  ggplot(aes(x = pac_cate, y = complex_pt_q)) +
  geom_density_ridges2(fill = "white") +
  geom_vline(xintercept = med_cate,
             color = "red", linetype = 2, size = 1) +
  ggtitle("A. Medical Complexity") +
  scale_y_discrete("Percent Medically Complex\nPatients (Quintiles)",
                   expand = expansion(mult = c(0, 0.2))) +
  scale_x_continuous("CATE Estimates", labels = dollar,
                     breaks = cate_breaks) +
  my_theme

complex_pat_ridge

## Picking joint bandwidth of 90.6
```

© 2022 Meath THA et al. *JAMA Health Forum.*

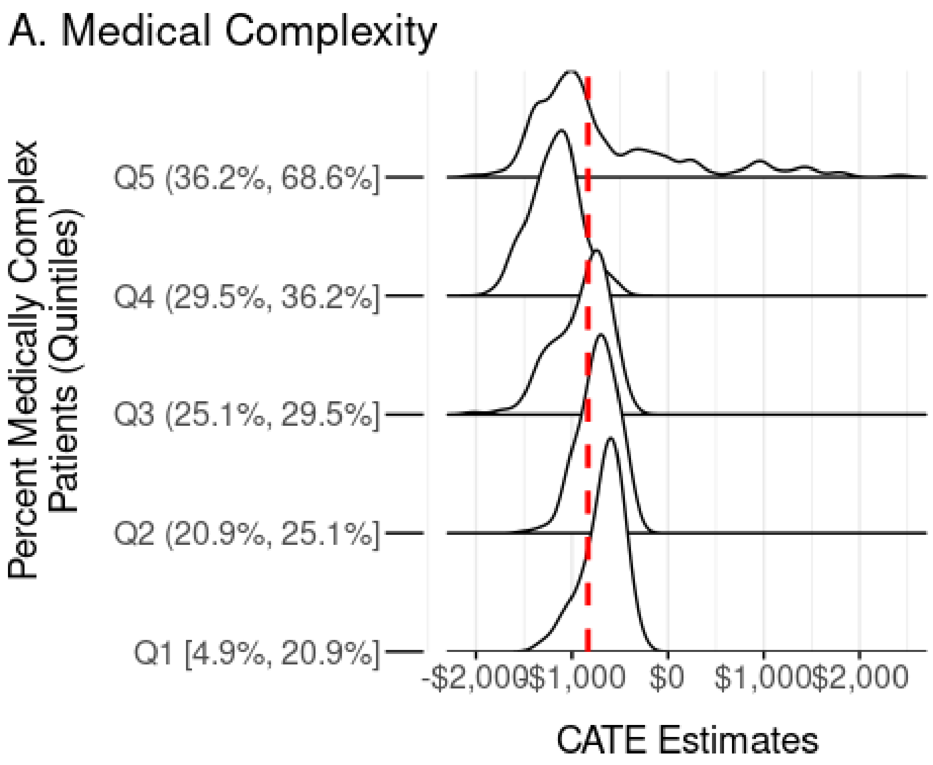

Stratified ATE by quintile

```
complex_pat_q <-
  quantiles_ate(pac_spending_cf,
                predictor = analytic$pct_complex_pt,
                quantiles = seq(0,1,0.2))
```

Plot

```
complex_pat_ate <-
  ggplot(complex_pat_q,
         aes(x = ate_est, xmin = ate_low, xmax = ate_high, y = q_label
)) +
  geom_point() +
  geom_errorbarh()  +
  geom_vline(xintercept = ate[[1]],
             color = "red", linetype = 2, size = 1) +
  ggtitle("A. Medical Complexity") +
  scale_y_discrete("Pre-period Medically Complex\nPatient Percentage (
Quintiles)",
                   expand = expansion(mult = c(0, 0.2))) +
  scale_x_continuous("ATE Estimates", labels = dollar,
                     breaks = ate_breaks) +
  my_theme

complex_pat_ate
```

© 2022 Meath THA et al. *JAMA Health Forum.*

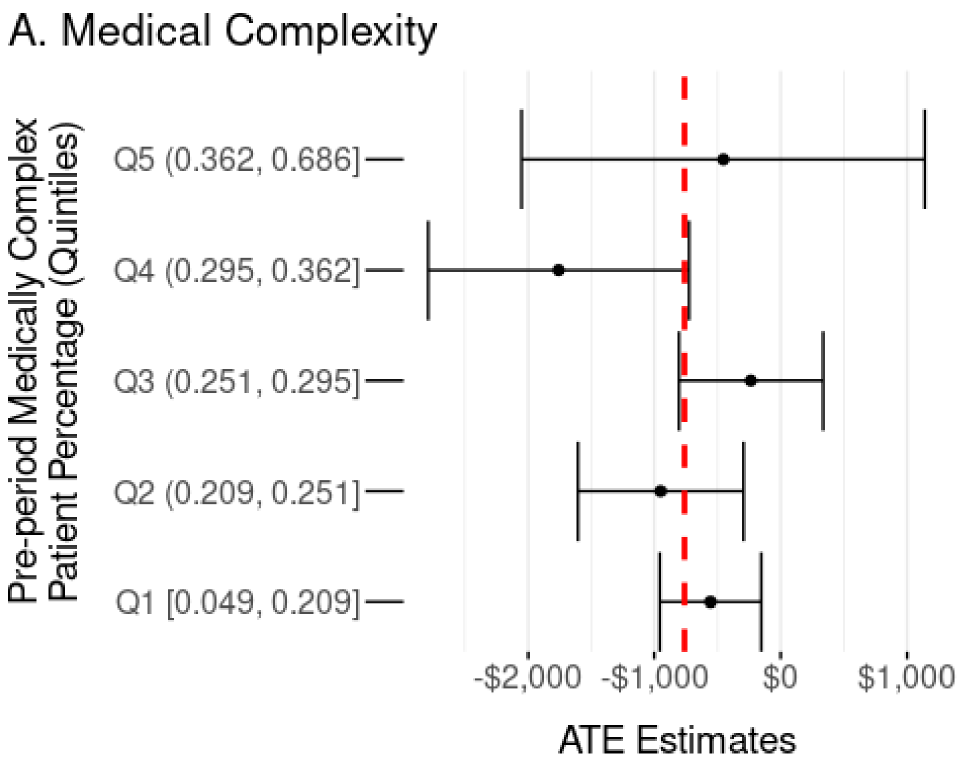

Predictor: Percent Dual-Eligible

Proportion of patients who are enrolled in both Medicaid and Medicare. These patients have lower SES and are generally more medically and socially complex.

Best Linear Projection

Test if predictor is linearly associated with DR scores (i.e. Treatment effect)

```
best_linear_projection(pac_spending_cf,
                       analytic$pct_dual)

##
## Best linear projection of the conditional average treatment effect.
## Confidence intervals are cluster- and heteroskedasticity-robust (HC
3):
##
##             Estimate Std. Error t value Pr(>|t|)
## (Intercept) -1047.97     320.66 -3.2682 0.001113 **
## A1           5482.01    4277.01  1.2817 0.200185
## ---
## Signif. codes:  0 '***' 0.001 '**' 0.01 '*' 0.05 '.' 0.1 ' ' 1
```

© 2022 Meath THA et al. *JAMA Health Forum.*

CATE Ridgeline by Quintile

```
analytic[, dual_q := fancy_quantile(pct_dual,
                                    quantiles = seq(0, 1, 0.2),
                                    label_func = percent_format(0.1))]
```

Next, the ridgeline plot

```
dual_ridge <-
  analytic %>%
  ggplot(aes(x = pac_cate, y = dual_q)) +
  geom_density_ridges2(fill = "white") +
  geom_vline(xintercept = med_cate,
             color = "red", linetype = 2, size = 1) +
  ggtitle("B. Social Complexity") +
  scale_y_discrete("Percent Socially Complex\nPatients (Quintiles)",
                   expand = expansion(mult = c(0, 0.2))) +
  scale_x_continuous("CATE Estimates", labels = dollar,
                     breaks = cate_breaks) +
  my_theme

dual_ridge

## Picking joint bandwidth of 102
```

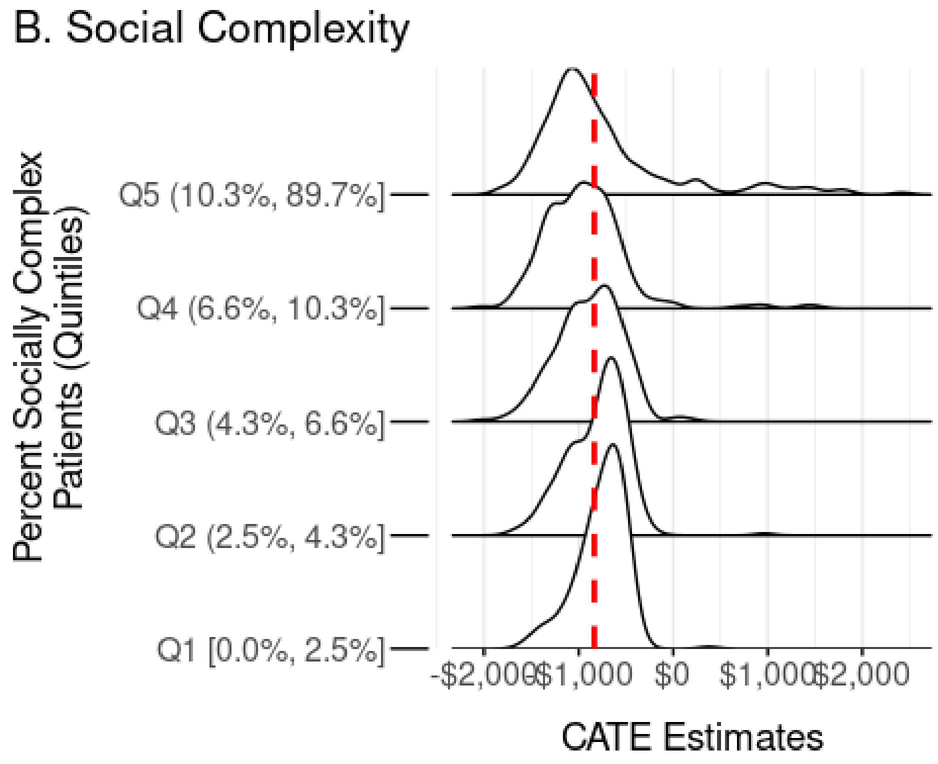

© 2022 Meath THA et al. *JAMA Health Forum.*

Stratified ATE by quintile

```
dual_quint <- quantiles_ate(pac_spending_cf,
                            predictor = analytic$pct_dual,
                            quantiles = seq(0,1,0.2))
```

Plot

```
dual_ate <-
  ggplot(dual_quint,
         aes(x = ate_est, xmin = ate_low, xmax = ate_high, y = q_label
)) +
  geom_point() +
  geom_errorbarh()  +
  geom_vline(xintercept = ate[[1]],
             color = "red", linetype = 2, size = 1) +
  ggtitle("B. Social Complexity") +
  scale_y_discrete("Pre-period Socially Complex\nPatient Percentage (Q
uintiles)",
                   expand = expansion(mult = c(0, 0.2))) +
  scale_x_continuous("ATE Estimates", labels = dollar,
                     breaks = ate_breaks) +
  my_theme

dual_ate
```

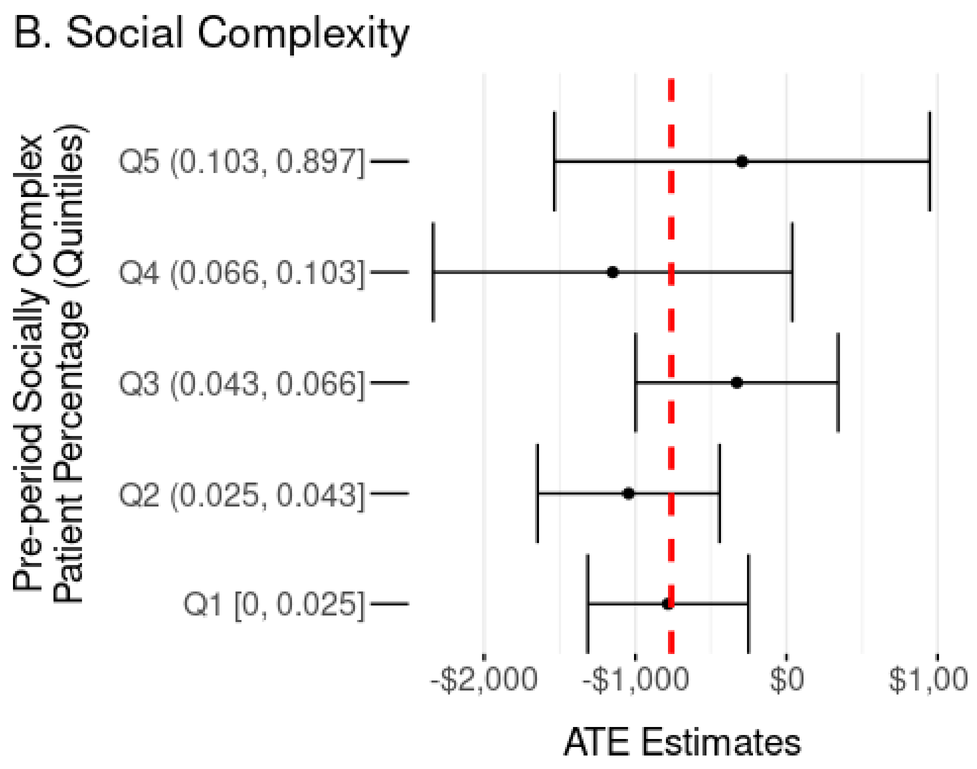

© 2022 Meath THA et al. *JAMA Health Forum.*

Predictor: LEJR Volume

Number of joint replacements provided in the pre-intervention period.

Best Linear Projection

Test if predictor is linearly associated with DR scores (i.e. Treatment effect)

```
best_linear_projection(pac_spending_cf, analytic$lejr_n)

##
## Best linear projection of the conditional average treatment effect.
## Confidence intervals are cluster- and heteroskedasticity-robust (HC
3):
##
##                Estimate  Std. Error t value Pr(>|t|)
## (Intercept) -785.493702  304.352870 -2.5809 0.009974 **
## A1             0.027862    0.237837  0.1171 0.906762
## ---
## Signif. codes:  0 '***' 0.001 '**' 0.01 '*' 0.05 '.' 0.1 ' ' 1
```

CATE Ridgeline by Quintile

```
analytic[, volume_q := fancy_quantile(lejr_n,
                                      quantiles = seq(0, 1, 0.2))]
```

Next, the ridgeline plot

```
volume_ridge <-
  analytic %>%
  ggplot(aes(x = pac_cate, y = volume_q)) +
  geom_density_ridges2(fill = "white") +
  geom_vline(xintercept = med_cate,
             color = "red", linetype = 2, size = 1) +
  ggtitle("C. Surgical Volume") +
  scale_y_discrete("Number of Pre-period\nLEJR Encounters (Quintiles)"
,
                   expand = expansion(mult = c(0, 0.2))) +
  scale_x_continuous("CATE Estimates", labels = dollar,
                     breaks = cate_breaks) +
  my_theme

volume_ridge

## Picking joint bandwidth of 98.8
```

© 2022 Meath THA et al. *JAMA Health Forum.*

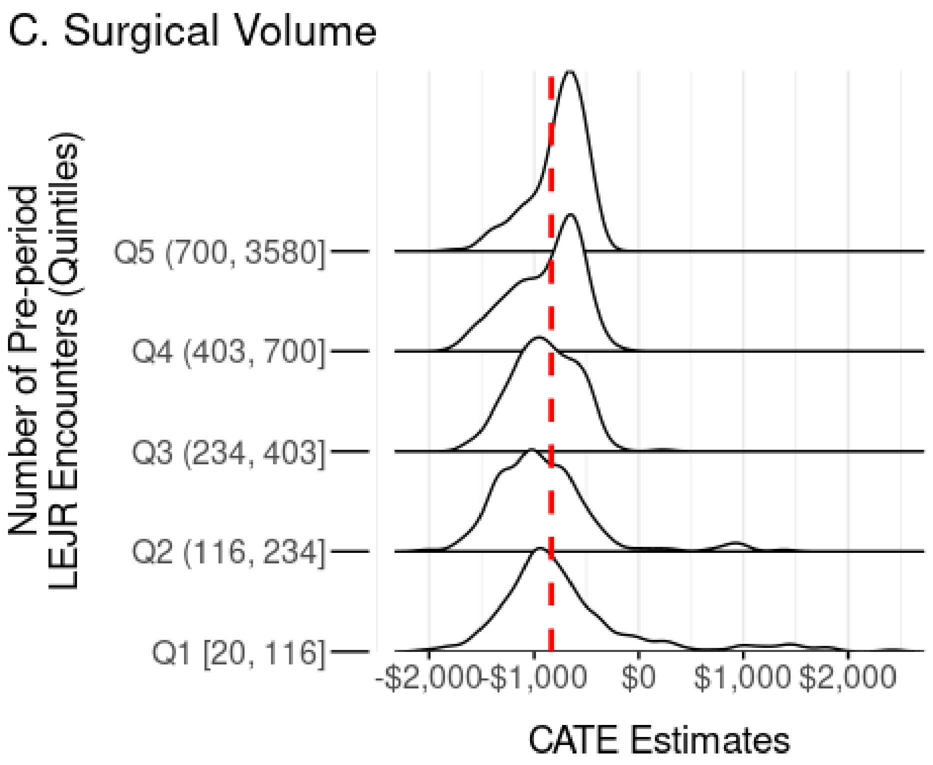

Stratified ATE by quintile

```
volume_quint <- quantiles_ate(pac_spending_cf,
                              predictor = analytic$lejr_n,
                              quantiles = seq(0,1,0.2))
```

Plot

```
volume_ate <-
  ggplot(volume_quint,
         aes(x = ate_est, xmin = ate_low, xmax = ate_high, y = q_label
)) +
  geom_point() +
  geom_errorbarh()  +
  geom_vline(xintercept = ate[[1]],
             color = "red", linetype = 2, size = 1) +
  ggtitle("C. Surgical Volume") +
  scale_y_discrete("Number of Pre-period\nLEJR Encounters (Quintiles)"
,
                   expand = expansion(mult = c(0, 0.2))) +
  scale_x_continuous("ATE Estimates", labels = dollar,
                     breaks = ate_breaks) +
  my_theme

volume_ate
```

© 2022 Meath THA et al. *JAMA Health Forum.*

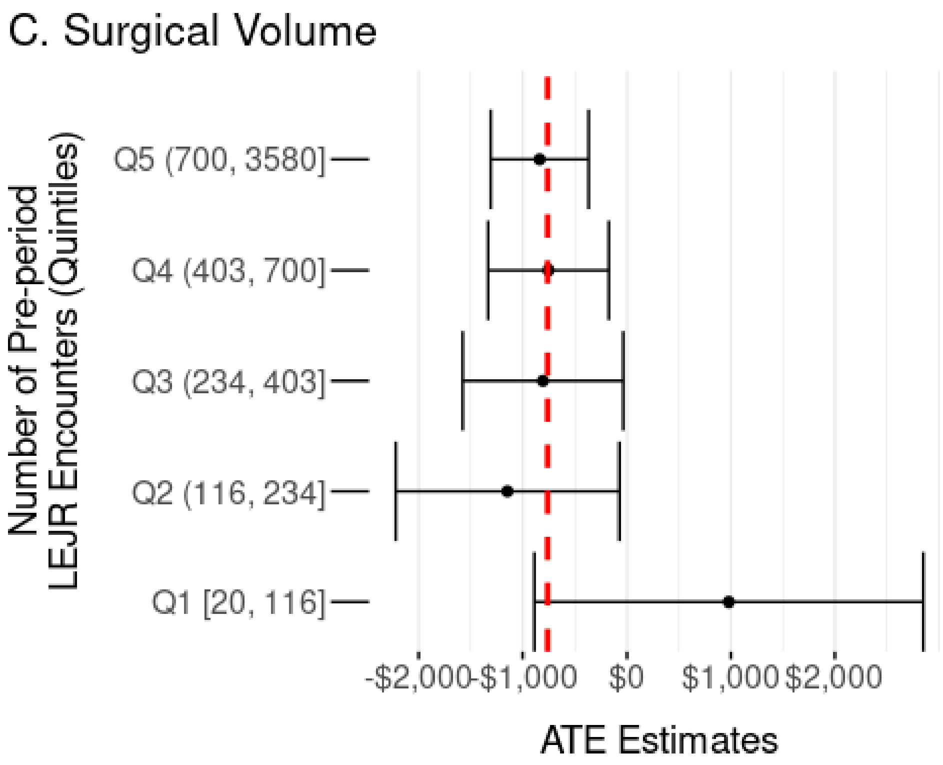

###Predictor: Pre-period PAC Spending###

Spending on PAC in the pre-period.

Best Linear Projection

Test if predictor is linearly associated with DR scores (i.e. Treatment effect)

```
best_linear_projection(pac_spending_cf,
                       analytic$pac_spending)

##
## Best linear projection of the conditional average treatment effect.
## Confidence intervals are cluster- and heteroskedasticity-robust (HC
3):
##
##                Estimate  Std. Error t value Pr(>|t|)
## (Intercept) -323.730697  470.979381 -0.6874   0.4920
## A1            -0.069570    0.087895 -0.7915   0.4288
```

CATE Ridgeline by Quintile

```
analytic[, pac_q := fancy_quantile(pac_spending,
                                   quantiles = seq(0, 1, 0.2),
                                   label_func = dollar_format(1))]
```

Next, the ridgeline plot

© 2022 Meath THA et al. *JAMA Health Forum.*

```r
pac_ridge <-
  analytic %>%
  ggplot(aes(x = pac_cate, y = pac_q)) +
  geom_density_ridges2(fill = "white") +
  geom_vline(xintercept = med_cate,
             color = "red", linetype = 2, size = 1) +
  ggtitle("D. Pre-period Institutional PAC Spending") +
  scale_y_discrete("Pre-Period Institutional Post-Acute\nCare Spending
(Quintiles)",
                   expand = expansion(mult = c(0, 0.2))) +
  scale_x_continuous("CATE Estimates", labels = dollar,
                     breaks = cate_breaks) +
  my_theme

pac_ridge

## Picking joint bandwidth of 94
```

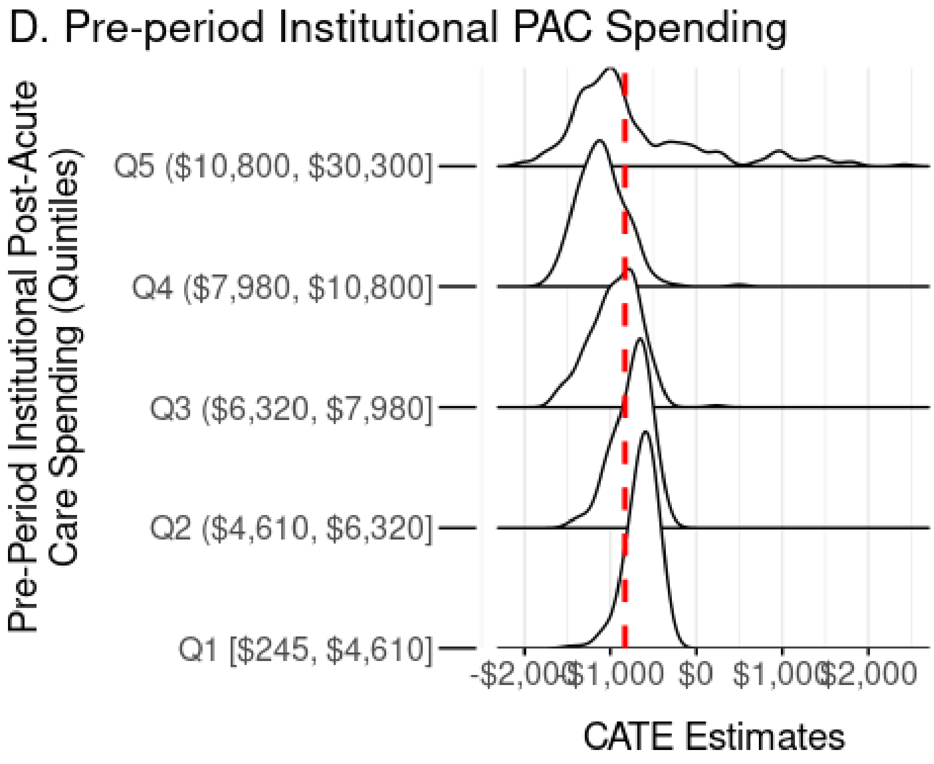

Stratified ATE by quintile

```r
pac_quint <- quantiles_ate(pac_spending_cf,
                           predictor = analytic$pac_spending,
                           quantiles = seq(0,1,0.2),
                           label_func = dollar_format(1))
```

Plot

© 2022 Meath THA et al. *JAMA Health Forum.*

```
pac_ate <-
  ggplot(pac_quint,
         aes(x = ate_est, xmin = ate_low, xmax = ate_high, y = q_label
)) +
  geom_point() +
  geom_errorbarh()  +
  geom_vline(xintercept = ate[[1]],
             color = "red", linetype = 2, size = 1) +
  ggtitle("D. Pre-period Institutional PAC Spending") +
  scale_y_discrete("Pre-Period Institutional Post-Acute\nCare Spending
(Quintiles)",
                   expand = expansion(mult = c(0, 0.2))) +
  scale_x_continuous("ATE Estimates", labels = dollar,
                     breaks = ate_breaks) +
  my_theme

pac_ate
```

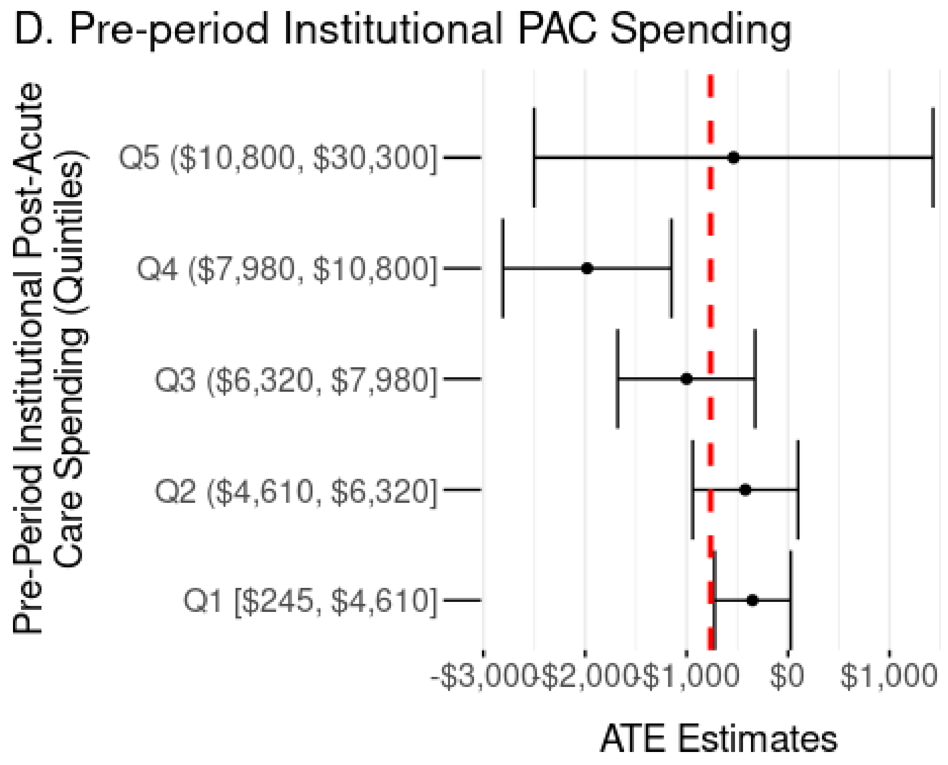

© 2022 Meath THA et al. *JAMA Health Forum.*

Save Output

CATE estimates

```
saveRDS(analytic[, .(provider, pac_cate)],
        file = file.path(inpath, "grf_cate_estimates.r"))
```

CATE Histogram

```
ggsave(filename = file.path(plot_path, "cate_hist.png"),
       plot = plot_cate,
       height = 5.5, width = 8, units = "in")

## Warning: Removed 2 rows containing missing values (geom_bar).
```

Ridgelines

```
ridges <-
  complex_pat_ridge +
  dual_ridge +
  volume_ridge +
  pac_ridge +
  plot_layout(ncol = 2, nrow = 2)

ggsave(filename = file.path(plot_path, "ridge_complex.png"),
       plot = complex_pat_ridge,
       height = 4.25, width = 5.5, units = "in")

## Picking joint bandwidth of 90.6

ggsave(filename = file.path(plot_path, "ridge_dual.png"),
       plot = dual_ridge,
       height = 4.25, width = 5.5, units = "in")

## Picking joint bandwidth of 102

ggsave(filename = file.path(plot_path, "ridge_volume.png"),
       plot = volume_ridge,
       height = 4.25, width = 5.5, units = "in")

## Picking joint bandwidth of 98.8

ggsave(filename = file.path(plot_path, "ridge_prepac.png"),
       plot = pac_ridge,
       height = 4.25, width = 5.5, units = "in")

## Picking joint bandwidth of 94

ggsave(filename = file.path(plot_path, "ridges.png"),
       plot = ridges,
       height = 8.5, width = 11, units = "in")
```

© 2022 Meath THA et al. *JAMA Health Forum.*

```
## Picking joint bandwidth of 90.6

## Picking joint bandwidth of 102

## Picking joint bandwidth of 98.8

## Picking joint bandwidth of 94
```

Stratified ATE

```
ate_strata <-
  complex_pat_ate +
  dual_ate +
  volume_ate +
  pac_ate +
  plot_layout(ncol = 2, nrow = 2)

ggsave(filename = file.path(plot_path, "complex_ate_ols.png"),
       plot = complex_pat_ate,
       height = 4.25, width = 5.5, units = "in")

ggsave(filename = file.path(plot_path, "dual_ate_ols.png"),
       plot = dual_ate,
       height = 4.25, width = 5.5, units = "in")

ggsave(filename = file.path(plot_path, "volume_ate_ols.png"),
       plot = volume_ate,
       height = 4.25, width = 5.5, units = "in")

ggsave(filename = file.path(plot_path, "prepac_ate_ols.png"),
       plot = pac_ate,
       height = 4.25, width = 5.5, units = "in")

ggsave(filename = file.path(plot_path, "ate_strata.png"),
       plot = ate_strata,
       height = 8.5, width = 11, units = "in")
```

© 2022 Meath THA et al. *JAMA Health Forum.*

Session Info

```
Run Length: 19.2 secs

R version 4.1.2 (2021-11-01)
Platform: x86_64-pc-linux-gnu (64-bit)
Running under: Red Hat Enterprise Linux

Matrix products: default
BLAS/LAPACK: /usr/lib64/libopenblas-r0.3.3.so

attached base packages:
[1] stats     graphics  grDevices utils     datasets  methods   base

other attached packages:
 [1] magrittr_2.0.2    tableone_0.13.0   rpart_4.1.16      scales_1.1.
1
 [5] dplyr_1.0.8       grf_2.0.2         patchwork_1.1.1   ggridges_0.
5.3
 [9] stringr_1.4.0     ggplot2_3.3.5     data.table_1.14.2

loaded via a namespace (and not attached):
 [1] Rcpp_1.0.8.3      lattice_0.20-45   class_7.3-19      zoo_1.8-9
 [5] assertthat_0.2.1  digest_0.6.29     lmtest_0.9-39     utf8_1.2.2
 [9] R6_2.5.1          plyr_1.8.7        labelled_2.9.0    survey_4.1-
1
[13] evaluate_0.15     e1071_1.7-9       highr_0.9         pillar_1.7.
0
[17] rlang_1.0.2       rstudioapi_0.13   Matrix_1.3-4      rmarkdown_2
.11
[21] textshaping_0.3.6 labeling_0.4.2    splines_4.1.2     munsell_0.5
.0
[25] proxy_0.4-26      compiler_4.1.2    xfun_0.29         pkgconfig_2
.0.3
[29] systemfonts_1.0.4 DiceKriging_1.6.0 htmltools_0.5.2   mitools_2.4
[33] tidyselect_1.1.2  tibble_3.1.6      fansi_1.0.3       crayon_1.5.
1
[37] withr_2.5.0       MASS_7.3-54       grid_4.1.2        gtable_0.3.
0
[41] lifecycle_1.0.1   DBI_1.1.2         cli_3.2.0         stringi_1.7
.6
[45] farver_2.1.0      ellipsis_0.3.2    ragg_1.2.2        generics_0.
1.2
[49] vctrs_0.3.8       sandwich_3.0-1    tools_4.1.2       forcats_0.5
.1
```

© 2022 Meath THA et al. *JAMA Health Forum.*

```
[53] glue_1.6.2        purrr_0.3.4       hms_1.1.1         fastmap_1.1
.0
[57] survival_3.2-13   yaml_2.3.5        colorspace_2.0-3  knitr_1.37
[61] haven_2.4.3
```

© 2022 Meath THA et al. *JAMA Health Forum.*
